# Plastome phylogenomics and morphological traits analyses provide new insights into the phylogenetic position, species delimitation and speciation of *Triplostegia* (Caprifoliaceae)

**DOI:** 10.1186/s12870-023-04663-4

**Published:** 2023-12-15

**Authors:** Qing-Li Fu, Zhi-Qiong Mo, Xiao-Guo Xiang, Richard I. Milne, Hans Jacquemyn, Kevin S. Burgess, Ya-Nan Sun, Hua Yan, Li Qiu, Bo-Yun Yang, Shao-Lin Tan

**Affiliations:** 1 Jiangxi Province Key Laboratory of Plant Resources, School of Life Sciences, Nanchang University, Nanchang, Jiangxi 330031 China; 2 CAS Key Laboratory for Plant Diversity and Biogeography of East Asia, Kunming Institute of Botany, Chinese Academy of Sciences, Kunming, Yunnan 650201 China; 3 Jiangxi Province Key Laboratory of Watershed Ecosystem Change and Biodiversity, Institute of Life Science, Nanchang University, Nanchang, Jiangxi 330031 China; 4 Institute of Molecular Plant Sciences, School of Biological Sciences, University of Edinburgh, Edinburgh, EH9 3JH UK; 5 KU Leuven, Department of Biology, Plant Conservation and Population Biology, B-3001 Leuven, Belgium; 6 College of Letters and Sciences, Columbus State University, University System of Georgia, Columbus, GA 31907-5645 USA

**Keywords:** Plastid genome, Phylogenomics, Speciation, Species delimitation, *Triplostegia*

## Abstract

**Background:**

The genus *Triplostegia* contains two recognized species, *T. glandulifera* and *T. grandiflora*, but its phylogenetic position and species delimitation remain controversial. In this study, we assembled plastid genomes and nuclear ribosomal DNA (nrDNA) cistrons sampled from 22 wild *Triplostegia* individuals, each from a separate population, and examined these with 11 recently published *Triplostegia* plastomes. Morphological traits were measured from herbarium specimens and wild material, and ecological niche models were constructed.

**Results:**

*Triplostegia* is a monophyletic genus within the subfamily Dipsacoideae comprising three monophyletic species, *T. glandulifera*, *T. grandiflora*, and an unrecognized species *Triplostegia* sp. A, which occupies much higher altitude than the other two. The new species had previously been misidentified as *T. glandulifera*, but differs in taproot, leaf, and other characters*. Triplotegia* is an old genus, with stem age 39.96 Ma, and within it *T. glandulifera* diverged 7.94 Ma. *Triplostegia grandiflora* and sp. A diverged 1.05 Ma, perhaps in response to Quaternary climate fluctuations. Niche overlap between *Triplostegia* species was positively correlated with their phylogenetic relatedness.

**Conclusions:**

Our results provide new insights into the species delimitation of *Triplostegia*, and indicate that a taxonomic revision of *Triplostegia* is needed. We also identified that either *rpoB-trnC* or *ycf1* could serve as a DNA barcode for *Triplostegia*.

**Supplementary Information:**

The online version contains supplementary material available at 10.1186/s12870-023-04663-4.

## Introduction

Accurate species delimitation plays a crucial role in assessing, monitoring, and conserving biodiversity [1–3]. This is made more difficult by cryptic species, defined as two or more distinct species that are erroneously classified under one species [4]. These may be morphologically barely distinguishable, yet on different evolutionary trajectories [4, 5]. These hidden or unrecognized species represent a substantial fraction of biodiversity, posing challenges to taxonomy, biodiversity estimation and conservation efforts [6–9]. Cryptic diversity can arise through various mechanisms [5, 10], such as recent divergence [11–13], convergent evolution driven by similar environmental pressures [14, 15], hybridization [6, 16, 17], and polyploidization [18, 19]. Over the past two decades, DNA barcodes (standard DNA regions across taxa) have been widely used in discriminating species [20–23] and discovering cryptic species [24, 25]. However, the standard plant DNA barcodes are not always effective, especially for recently radiated taxa or those possess complex evolutionary histories [26, 27].

The plastid genome in almost all land plants exhibits a highly conserved quadripartite structure [28], generally ranging from 120 to 160 kb in size and containing 110–130 distinct genes including ~ 80 protein-coding genes, 30 transfer RNA (tRNA) genes, and four ribosomal RNA (rRNA) genes [29, 30]. Plastid genomes are predominantly maternally inherited in plants [31]. Due to their high copy number within cells, plastid genomes can be sequenced, assembled, and annotated more easily and cost-effectively than nuclear genomes [32, 33], aiding their widespread use in elucidating the evolutionary history of green plants [34–36]. Moreover, plastid genomes have emerged as super DNA barcodes or ultra-barcodes [37, 38], containing a higher number of informative sites and exhibiting greater discriminatory power than standard plant DNA barcodes [39, 40]. Plastid genomes have recently been used in discovering cryptic species and screening taxon-specific DNA barcodes for particular plant lineages [19, 41, 42].

The Hengduan Mountains Region (HDM), also known as the Mountains of Southwest China, is recognized as one of the world’s biodiversity hotspots [43, 44]. It is known for harboring the richest temperate alpine flora in the world [45], and as a center of diversity for numerous plant lineages [46–49]. Its topography is characterized by a series of north-south oriented alpine mountain ranges separated by deep river gorges [47], which act as genetic barriers for some plant taxa [50, 51] and therefore have contributed to the high species diversity in this region.

**Fig. 1:**
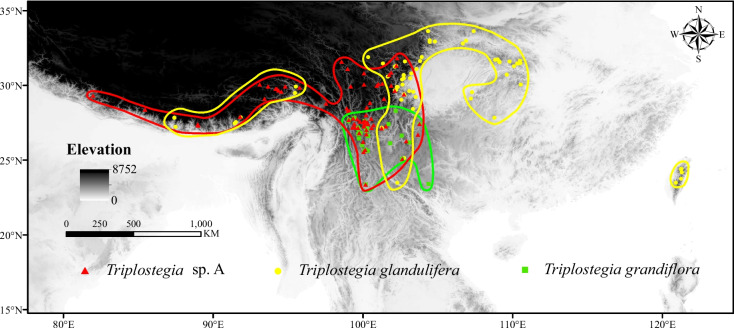
Occurrences of *Triplostegia glandulifera* (yellow dot), *Triplostegia* sp. A (red triangles), and *T. grandiflora* (green squares). The areas circled with yellow, red, and green lines are the geographical distribution areas of *T. glandulifera*, *Triplostegia* sp., and *T. grandiflora*, respectively. (The map is created by authors using ArcGIS software)

The genus *Triplostegia* Wall. ex DC. comprises two traditionally recognized species: *T. grandiflora* Gagnep. (1901) is confined to the HDM in north Yunnan and West Sichuan, whereas *T. glandulifera* Wall. ex DC. (1830) is widely distributed in the mountains of southwestern and central China, extending to Taiwan, Bhutan and Nepal [52] (Fig. 1). Recent evidence placed *Triplostegia* within subfamily Dipsacoideae of Caprifoliaceae [53–55], but its affinities have long been controversial [56–58]. Morphologically, *Triplostegia grandiflora* differs from *T. glandulifera* in its sessile (not petiolate) leaves, longer corolla and more elongated inflorescence branch [52], but recent studies based on molecular and morphological evidence have proposed merging *T. grandiflora* into *T. glandulifera* [59, 60]. However, our own investigations in the southern region of the HDM have revealed a third taxonomic entity, here termed *Triplostegia* sp. A, which often occurs sympatrically with *T. grandiflora*, but differs from both recognized species in glabrous and slender lateral taproot, petiolate and marginal serrated leaves, and corollas usually 1–2 mm in length. Furthermore, whereas *T. grandiflora* exists in *Pinus yunnanensis* and *P. armandii* forests up to 2066–3128 m, *Triplostegia* sp. A has a larger elevation range, 2651–3954 m according to our fieldwork, occurring in *Pinus*, *Quercus*, *Abies*, and *Picea* forests plus roadsides, riversides, and alpine meadows.

In this study, we performed genome skimming on 22 individuals collected from 14 populations of *Triplostegia* sp. A and eight populations of *T. grandiflora*, all from the southern HDM within the northwest region of the Yunnan Province. To these were added 11 recently published *Triplostegia* plastomes covering the entire distribution range of *Triplostegia* [59]. Furthermore, we recorded morphological and functional traits of *Triplostegia* species from herbarium specimens and wild plants. Our main objectives were to address the following questions. (1) What is the phylogenetic position of *Triplostegia*? (2) How many distinct species exist within *Triplostegia*? (3) When did diversification occur among *Triplostegia* species? (4) Are there any highly variable regions in the plastid genome that could be used as taxon-specific DNA barcodes for discriminating *Triplostegia* species? (5) Did any geographical features play a role promoting diversification within *Triplostegia*?

## Materials and methods

### Taxon sampling

One individual was randomly selected from each of 8 and 14 populations of *T. grandiflora* and *Triplostegia* sp. A in northwest Yunnan Province, respectively (Table S1; Fig. 6). Healthy and fresh leaves were collected and immediately dried using silica gel. Vouchers were deposited in the Herbarium of Nanchang University. In addition, 11 sequences of *Triplostegia* were downloaded from the NCBI Sequence Read Archive (SRA) for analysis, comprising seven samples of *T. glandulifera*, one of *T. grandiflora*, and three of the unrecognized species *Triplostegia* sp. A (Table S1).

### DNA isolation and sequencing

Total genomic DNA was extracted from silica-gel-dried leaves using a modified CTAB method [61]. The DNA samples were then sheared into fragments and used to construct 500 bp libraries by the Molecular Biology Experiment Center, Germplasm Bank of Wild Species in Southwest China, following the manufacturer’s manual (Illumina, San Diego, CA, USA). Paired-end sequencing of 150 bp was performed on an Illumina HiSeq 2500 platform.

### Plastid genome and nrDNA assembly and annotation

The raw sequence reads were quality-checked with FastQC [62] and filtered using Trimmomatic v0.3.2 [63]. The plastid genomes and nrDNA sequences were assembled from high-quality paired reads using GetOrganelle v1.6.0 [64] with default settings. The connections between contigs were evaluated and visualized using Bandage v0.7.1 [65]. The assembled plastid genomes were annotated using Geseq (MPI-MP CHLOROBOX - GeSeq(mpg.de)) [66], followed by manual adjustments in Geneious v9.05 (http://www.geneious.com/) using the published plastid genome of *T. glandulifera* (GenBank accession: NC_045051) as a reference. The assembled nrDNA sequences were annotated in Geneious using the nrDNA of *Scabiosa canescens* Waldst. & Kit. (MT735330) as a reference. Finally, the graphical maps of the *Triplostegia* plastid genomes were generated using Organellar Genome DRAW v. 1.3.1 [67].

### Genome comparison and structural analysis

The plastid genomes were aligned and visualized using mVISTA in Shuffle-LAGAN mode [68]. To investigate potential rearrangements in the plastid genomes, multiple sequence alignment was performed using MAUVE [69]. Comparisons of boundaries between the single-copy regions and the inverted repeat (IR) regions among the plastid genomes were performed using IRscope [70].

### Nucleotide diversity and genetic differentiation analysis

We used nucleotide diversity (π) to assess the levels of plastid genomic divergence within *Triplostegia*, and identify highly variable plastid DNA regions, using DNAsp v6.0 [71], employing a window length of 600 bp and a step size of 200 bp.

Genetic differentiation (*F*_ST_) and gene flow (*N*_m_) among species, as well as within-species genetic diversity, were also estimated using DNAsp v6.0 [71], with all samples belonging to each taxon being considered a population.

### Phylogenetic analysis

Phylogenetic relationships among the 33 *Triplostegia* samples (Table S1) were examined using Maximum-likelihood analysis (ML) and Bayesian inference (BI), based on three datasets: the complete plastid genomes, plastid protein-coding sequences (CDS), and nrDNA sequences. As outgroups we used *Pterocephalus hookeri* (C.B.Clarke) Airy Shaw & M.L.Green, *Dipsacus asper* Wall. ex DC., *Scabiosa tschiliensis* Grüning, *Kolkwitzia amabilis* Graebn., *and Patrinia heterophylla* Bunge, representing genera closely related to *Triplostogia* [59]*.* Three samples were randomly selected from each of the three clades formed by the 33 samples of *Triplostegia*, and used to determine the position of the genus in the phylogeny of Dipsacales via ML and BI analyses. For this, 57 complete plastid genomes were obtained, including the above outgroups and covering of the recognized families within the order (Table S2). *Sesamum indicum* L*.*, *Mentha spicata* L*.*, *Pittosporum kerrii* Craib, and *Apium graveolens* L*.* were chosen as outgroups to Dipsacales based on previous studies [53–55].

Multiple sequence alignments were performed using MAFFT v7.409 [72]. The best-fit nucleotide substitution model was selected using ModelTest v.3.7 [73] with the Akaike information criterion (AIC). ML analysis was performed using RAxML v 8.2.12 [74] under the GTRGAMMA model with 1000 bootstrap replicates. BI analysis was performed using MrBayes v3.2.6 [75] with the Markov Chain Monte Carlo (MCMC) algorithm, running for 2,000,000 generations with the first 25% of trees discarded as burn-in, and thereafter sampling trees every 1000 generations, and using these to construct majority-rule consensus trees. Furthermore, phylogenetic networks of plastid genomes and nrDNA sequences were visualized using SplitsTree v4.14.6 [76].

### Species discrimination analysis

We assessed the effectiveness of standard plant DNA barcodes, including *rbcL*, *matK*, and ITS, and the barcode *ycf1* suggested by Dong et al. (2015) [77], plus the highly variable plastid DNA regions of *Triplostegia* and their combinations, in discriminating *Triplostegia* species using tree-based methods. ML trees for each marker were constructed using RAxML with the same settings as previously described. A species was considered as being correctly resolved when all the individuals of the same species formed a monophyletic group with >70% bootstrap support [78]. The standard DNA barcode *trnH-psbA* was not included in the analysis due to its insufficient number of informative sites for species discrimination. In addition, we generated Neighbour-Joining (NJ) trees using MEGA v10 [79] based on the highly variable plastid DNA regions and the ITS region, using the P-distance model with 1000 bootstrap replicates.

In addition to the tree-based analyses, we also conducted distance-based analyses following Hollingsworth et al. (2009) [22]. Pairwise interspecific and intraspecific genetic distances were calculated using the Kimura 2-parameter (k2p) mode using MEGA v10 [79]. A species was considered to be successfully discriminated if its minimum interspecific k2p distance involving this species was greater than its maximum intraspecific k2p distance.

### Divergence time estimation

We obtained plastome sequences from GenBank (Table S3) for a total of 27 species of Dipsacales, two species of Apiales (*Apium graveolens* L. and *Pittosporum kerrii* Craib), and two species of Lamiales (*Sesamum indicum* L. and *Mentha spicata* L.) for the purpose of estimating divergence times. ModelTest analysis indicated that the GTR + I + G nucleotide substitution model performed the best (Table S4). We used BEAST v2.6.6 [80] to estimate divergence times under a relaxed lognormal clock and GTR + I + G nucleotide substitution model. Markov Chain Monte Carlo (MCMC) searches were performed for 500,000,000 generations, sampling every 25,000 generations. The tree prior was specified as a Yule process. Tracer v.1.5 [81] was used to assess chain convergence and to ensure that the effective sample sizes (ESS) were greater than 200. The maximum clade credibility (MCC) tree with median heights was computed using TreeAnnotator v2.6.6. Four calibration points were used: (1) the crown age of Dipsacales was set to 103 million years ago (Ma), with a normal prior (mean = 103 Ma, SD = 1.0), based on previous studies [82, 83]; (2) the earliest fossil record of *Viburnum* from the late Paleocene to early Eocene [84, 85] was used to calibrate the crown group of Adoxaceae, with lognormal prior (mean = 0, SD = 1.0, offset = 56 Ma), following Moore and Donoghue (2007) [86]; (3) the setting of divergence time between *Weigela* and its sister group *Diervilla*, with lognormal prior (mean = 0, SD = 1.0, offset = 23 Ma), following Wang et al., (2015) [83]; and (4) the fossil fruits of *Diplodipelta* (36 Ma) [87] were used to calibrate the stem age of *Dipelta*, with lognormal prior (lognormal mean = 0, SD = 1.0, offset = 36 Ma), following Wang et al., (2015) [83].

### Morphological and functional traits analyses

We collected morphological trait data of *Triplostegia* species by measuring specimens across their distribution range. For 63, 27, and 55 specimens of *T. glandulifera*, *T. grandiflora*, and *Triplostegia* sp. A respectively, we measured 10 morphological traits, including plant height, taproot length and width, leaf length and width, petiole length, leaf fission depth, corolla length, fruit length and width. These traits were chosen because most of them showed disparities among the *Triplostegia* species according to our observations. In addition, respectively 121 and 149 individuals from 8 and 14 populations of *T. grandiflora*, and *Triplostegia* sp. A (identified by S-L Tan) where they co-occur in northwest Yunnan (Fig. 6), were examined for eight morphological and functional traits: plant height, leaf chlorophyll content, leaf area, leaf thickness, leaf dry mass, specific leaf area (SLA), corolla length, and corolla width, following previously applied protocols [88–90]. We conducted Principal component analysis (PCA) based on these morphological traits. Kruskal-Wallis tests and pairwise Wilcoxon rank sum tests were used to assess the differences in each trait among the three *Triplostegia* taxa. All statistical analyses were conducted using R version 4.1.3 [91].

### Species distribution modelling

We used MaxEnt v.3.4.1 [92] to assess the suitable climate envelopes of *T. glandulifera*, *T. grandiflora*, and *Triplostegia* sp. A across the past, present, and future periods. Species occurrence records were obtained from the Chinese Virtual Herbarium (http://www.cvh.ac.cn/), the Global Biodiversity Information Facility (https://www.gbif.org/), plus our own field collections. To ensure data quality, we refined the occurrence records following the criteria described by Qiu, et al. (2023) [93] by removing: 1) duplicate records, 2) records lacking spatial coordinates or specific locations, 3) specimens with identification errors, and 4) unreliable records that were located in the city or bodies of water. To reduce the effect of spatial autocorrelation and the consequent overfitting, occurrence records within five kilometers of another were filtered out. Ultimately, our final dataset for species distribution modelling consisted of 64, 30, and 67 occurrence records for *T. glandulifera, T. grandiflora*, and *Triplostegia* sp. A, respectively (Fig. 1).

Nineteen bioclimatic variables were downloaded from WorldClim v1.4 for the Last Interglacial (LIG; 120,000–140,000 years ago), the Last Glacial Maximum (LGM; 22,000 years ago), and the Mid-Holocene (MH; 6000 years ago) periods, and from WorldClim v2.1 (https://www.worldclim.org/) for the present (1970–2000) and future (2090: average of 2081–2100) (Table S5), with a resolution of 2.5 arc-minute (approximately 5 km^2^). To provide a conservative and a comparatively larger estimate of species distribution change under future climate conditions, we used two Shared Socioeconomic Pathways (SSPs) for future climatic conditions: SSP2–4.5 (moderate climate change) and SSP5–8.5 (pessimistic climate change) from the CMIP6 (BCC-CSM2-MR) climate model [94]. To avoid multicollinearity of variables, we performed a Pearson’s correlation test for the 19 bioclimatic variables for each species, and for any pair of variables with Pearson’s *r* > 0.8, the variable with the higher percentage contribution was retained. The Area Under Receiver-Operating Characteristic (ROC) Curve (AUC) values were used to evaluate the accuracy of the species distribution models [95]. The AUC values range from 0.5 to 1, which are categorized as failing (0.5–0.6), poor (0.6–0.7), fair (0.7–0.8), good (0.8–0.9), and excellent (0.9–1) [96]. The Jackknife analysis was used to determine the relative significance of each bioclimatic variable [97]. To determine the potential distribution of each species, we reclassified the MaxEnt output file using the 10-percentile training presence logistic threshold value (10TPL) [98, 99]. We used the SDM toolbox v2.4 in ArcGIS 10.2 to calculate the suitable area changes between different periods.

To quantify niche similarity between species, we used the software ENMTools v1.3 [100] to estimate the niche overlap among *Triplostegia* species using two metrics: Schoener’s *D* [101] and Warren’s *I* [102]. Both metrics range from 0 to 1, with values closer to 1 indicating a higher degree of niche overlap between the species.

## Results

### General features of the Triplostegia plastid genomes

The 33 plastid genomes of *Triplostegia* examined (Table S1) were highly conserved in gene content, gene order, and GC content (Table 1; Fig. S1), all exhibiting a typical quadripartite structure composed of a large single-copy (LSC) region, a small single-copy region (SSC), and two inverted repeat regions (IR) (Fig. S1). The plastid genome lengths varied as follows: 154,230–155,445 bp for *T. glandulifera*, 155,041–155,410 bp for *Triplostegia* sp. A, and 155,638–155,706 bp for *T. grandiflora* (Table 1). The complete plastid genome consists of 113 unique genes, including 79 protein coding genes, 30 tRNA genes, and four rRNA genes. Among these, the following17 genes were found to be duplicated in the IR regions: *rrn16*, *rrn23*, *rrn4.5*, *rrn5*, *trnI-CAU*, *trnL-CAA*, *trnV-GAC*, *trnI-GAU*, *trnA-UGC*, *trnR-ACG*, *trnN-GUU*, *rps12*, *rps7*, *rpl23*, *ndhB*, *ycf2*, and *ycf1*. Additionally, 16 genes contained one intron (*trnK-UUU*, *trnL-UAA*, *trnV-UAC*, *trnI-GAU*, *trnA-UGC*, *rps16*, *rps12*, *rpl16*, *rpl2*, *rpoC1*, *petB*, *petD*, *atpF*, *ndhB*, *ndhA*, *ycf2*), whereas two genes (*clpP* and *ycf3*) contained two introns (Table S6).

**Table 1:** General characteristics of chloroplast genome of *Triplostegia* species

Species	Size (bp)	LSC (bp)	SSC (bp)	IR (bp)	Total CG (%)	Total Genes	Protein Genes	tRNA Genes	rRNA Genes
*T. glandulifera*	154,230–155,445	88,183–89,072	17,927–17,962	23,909–24,224	38.5–38.6	113	79	30	4
*Triplostegia* sp. A	155,041–155,410	88,795–88,963	17,831–17,840	24,200–24,329	38.5	113	79	30	4
*T. grandiflora*	155,638–155,706	89,005–89,073	17,599	24,517	38.5	113	79	30	4

*IR* Inverted repeat, *LSC* Long single-copy, *SSC* Short single-copy

### Comparative analysis of plastid genome structures

According to mVISTA (Fig. S2) and MAUVE (Fig. S3) analysis, the plastid genome structures were highly conserved in *Triplostegia*, with no inversion or rearrangement detected. The LSC and SSC regions were more variable than the IR regions, and the non-coding regions were more variable than the coding regions. The IR/SSC and IR/LSC junction regions of *Triplostegia* contained seven genes: *rpl2*, *rpl23*, *trnN*, *ndhF*, *ycf1*, *trnI*, and *trnH* (Fig. S4).

### Phylogenetic analyses and divergence time estimation

**Fig. 2:**
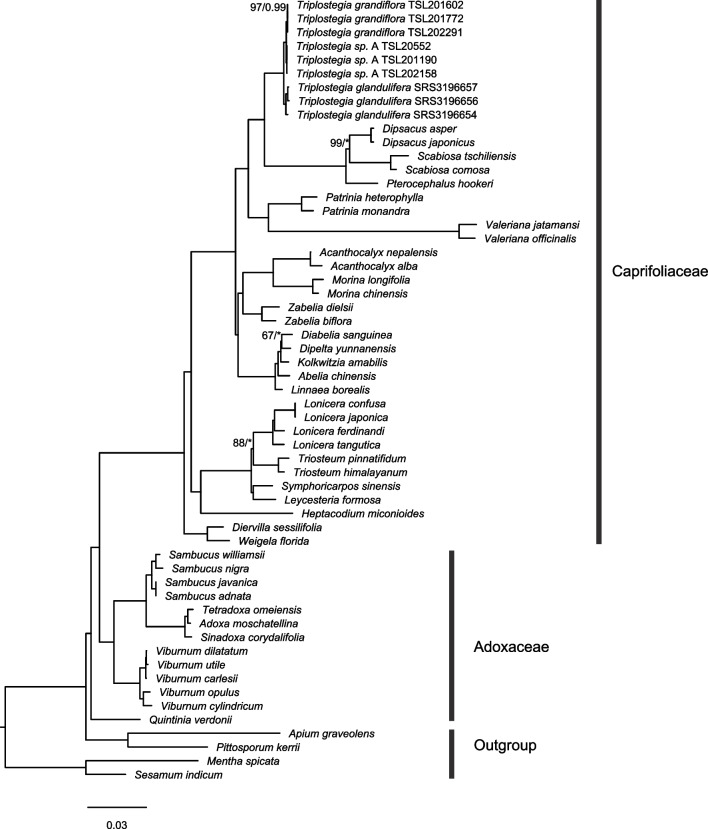
Phylogenetic relationships of Dipsacales constructed using RAxML based on complete chloroplast genome sequences. The maximum likelihood (ML) tree is presented, with maximum likelihood bootstrap support values (BS) and Bayesian inference posterior probability (PP) values given for each node. Nodes with a ‘*’ symbol represent nodes that received maximum support from ML or BI analysis (‘*’: 100% or 1.0). Nodes without values represent maximal support in both ML and BI methods (BS_ML_ = 100%, PP_BI_ = 1.00)

**Fig. 3:**
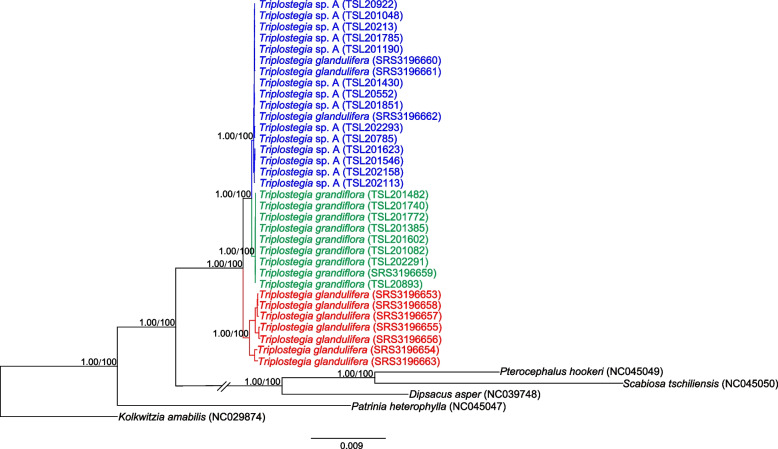
Phylogenetic relationships of 33 samples of *Triplostegia* species based on complete chloroplast genome sequences. The phylogenetic tree was constructed using both maximum likelihood (ML) and Bayesian inference (BI) methods. The maximum likelihood (ML) tree is presented. Numbers along the branch indicate bootstrap support values from ML analysis (based on 1000 replicates) and Bayesian posterior probabilities from BI analysis

**Fig. 4:**
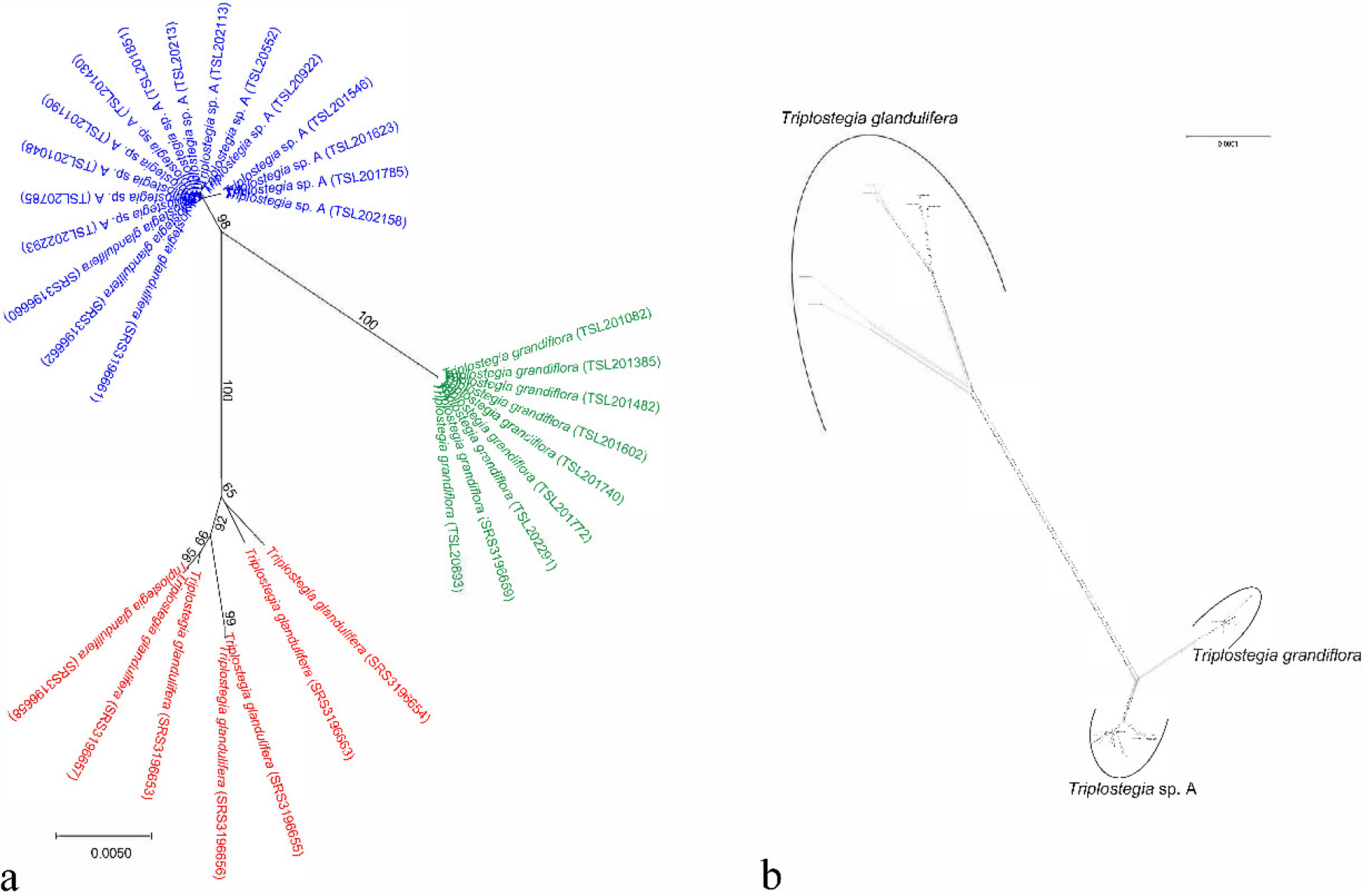
**a** Unrooted neighbour-joining (NJ) tree of *Triplostegia* based on the P-distance calculated from three highly variable plastid DNA regions (*ndhf*, *ndhf-trnN*, *rpoB-trnC*) and nuclear ITS sequences. **b** Neighbor-net analysis of *Triplostegia* based on complete chloroplast genome and nrNDA sequences. Bootstrap values (based on 1000 replicates) are indicated along the branches for each clusters

Our phylogenetic analyses of Dipsacales based on plastid genomes revealed that *Triplostegia* is a strongly supported (BS_ML_ = 100%, PP_BI_ = 1.00) monophyletic genus within the subfamily Dipsacoideae (Caprifoliaceae), forming a sister clade to a group consisting of *Dipsacus*, *Scabiosa*, and *Pterocephalus* (Fig. 2). Within *Triplostegia*, ML and BI analysis of both complete plastid genomes (Fig. 3) and plastid CDS (Fig. S5) all resolved three well-supported clades, corresponding to *T. glandulifera*, *T. grandiflora*, and *Triplostegia* sp. A. The phylogeny based on nrDNA sequences (Fig. S6) was similar except that of *T. glandulifera* did not form a monophyletic clade (Fig. S6). The three taxa also formed distinct clusters according to Neighbor-net analysis of concatenated complete plastid genomes and nrDNA sequences, with *T. glandulifera* displaying high levels of intraspecific genetic variation (Fig. 4).

**Fig. 5:**
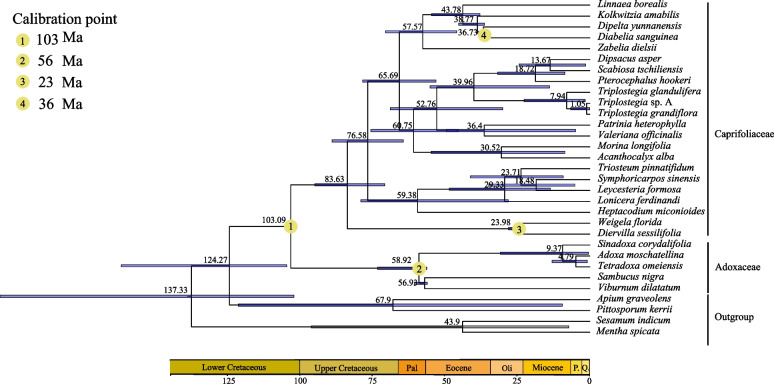
BEAST analysis of divergence times based on protein coding region sequences of the chloroplast genome. Calibration points are indicated by yellow circle. The blue bars indicate the 95% highest posterior density (HPD) credibility intervals for node ages

Molecular dating analysis (Fig. 5) estimated the stem and crown ages of *Triplostegia* to be 39.96 Ma (95% highest potential density, HPD: 13.91–55.05), and 7.94 Ma (95% HPD: 1.59–22.68) respectively, with the first diverging taxon being *T. glandulifera*, with *T. grandiflora* diverging from *Triplostegia* sp. around 1.05 Ma (95% HPD: 0.028–6.58).

### Nucleotide diversity and genetic differentiation

Among the three *Triplostegia* species, *T. glandulifera* displayed the highest plastomes genetic diversity (π = 1.17 × 10^−3^), with that of *Triplostegia* sp. A around eight times lower (π = 1.4 × 10^−4^), and *T. grandiflora* ~ seven time lower again (π = 2 × 10^−5^).

A total of 814 polymorphic loci were identified in the plastomes of *Triplostegia*, with average π values of 0.00146, 0.00246, and 0.00059 for the LSC, SSC, and IR regions, respectively (Fig. S7). Three highly variable regions were detected: *ndhF* (π = 0.03212) in the SSC region, *trnN-ndhF* (π = 0.02944) in the IRb region, and *rpoB-trnC* (π = 0.00682) in the LSC region.

**Fig. 6:**
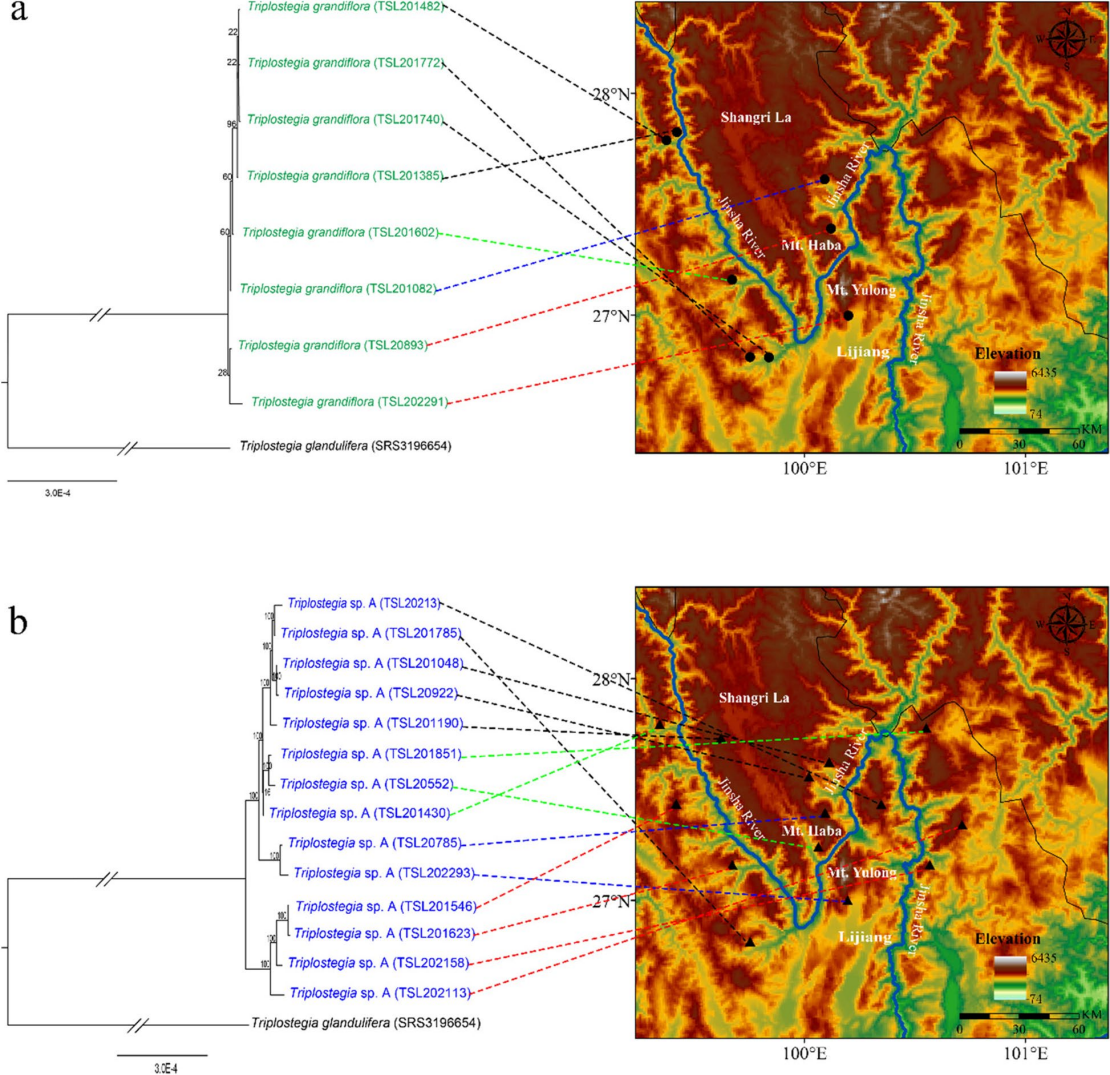
Correlations between phylogenetic relatedness and geographical distributions of samples from *T. grandiflora* (**a**) and *Triplostegia* sp. (**b**) in the alpine-gorge region of the Great Two Bends of Jinsha River. The phylogenetic relationships were inferred using the maximum likelihood (ML) method based on complete chloroplast genome sequences. (The maps are created by authors using ArcGIS software)

For both the plastid and nrDNA data, the degree of genetic differentiation (*F*_ST_), was relatively high among the three *Triplostegia* species (Table 2). *F*_ST_ was highest between *T. grandiflora* and the *Triplostegia* sp. A (0.89533 for plastids, 0.93251 for nrDNA), followed by *T. grandiflora* vs. *T. glandulifera* (0.80408 for plastids, 0.78292 for nrDNA), and *Triplostegia* sp. vs. *T. glandulifera* (0.77473 for plastids, 0.65421 for nrDNA). However, our phylogenetic analyses, based on plastid genomes, detected no correlation between the phylogenetic relatedness of samples and their geographical distribution on either the same or opposite sides of the Jinsha River, for both *T. grandiflora* and *Triplostegia* sp. (Fig. 6).

**Table 2:** Genetic differentiation (*F*_ST_) and gene flow (*N*_m_) among the three *Triplostegia* species based on complete plastid genomes and nrDNA sequences*.* All the samples of each species are regarded as one population

Population 1	Population 2	*F*_ST_		*N*_m_	
		plastid genome	nrDNA	Plastid genome	nrDNA
*T. grandiflora*	*Triplostegia* sp. A	0.89533	0.93251	0.029	0.018
*T. grandiflora*	*T. glandulifera*	0.80408	0.78292	0.061	0.069
*Triplostegia* sp. A	*T. glandulifera*	0.77473	0.65421	0.073	0.132

*N*_m_ = (1 - *F*_ST_) / 4 *F*_ST_

### Species discrimination based on standard DNA barcodes and highly variable cpDNA regions

In tree-based analyses, none of the standard plant DNA barcodes (*rbcL*, *matK*, *trnH-psbA*, and ITS), whether used singly or in combinations, could successfully discriminate all three *Triplostegia* taxa (Table 3)*.* However, the highly variable cpDNA region *rpoB-trnC* region alone successfully discriminated all three species with relatively high node support values (100% for *T. glandulifera*, 87% for *T. grandiflora*, and 98% for *Triplostegia* sp.), and the *ycf1* gene did the same. The highly variable cpDNA regions *ndhF* and *ndhF-trnN* could not distinguish all three *Triplostegia* species alone or in combination*.* All other combinations including two to four regions of *ndhF*, *ndhF-trnN*, *rpoB-trnC* and ITS, could successfully discriminate all three *Triplostegia* taxa except for *ndhF* + *ndhF-trnN* and *ndhF-trnN* + ITS. Particularly, the combination of *rpoB-trnC* and ITS was able to successfully discriminate all three *Triplostegia* species with maximum supporting values. Furthermore, all *T. grandiflora* samples contained an identical 66 bp insertion sequence in their *ycf1* gene, while all *T. glandulifera* samples contained an identical 18 bp insertion sequence in *ycf1*. Distance-based analyses revealed that any of the three highly variable plastid regions or the *ycf1* gene alone successfully distinguished all three *Triplostegia* species (Table S7).

**Table 3:** Tree-based species discrimination rates of *Triplostegia* by using highly variable plastid DNA regions, *ndhF*, *ndhF-trnN*, *rpoB-trnC*, and standard plant DNA barcodes, *rbcL*, *matK*, and ITS singly or in combinations

DNA regions or their combinations	Bootstrap values of monophyletic clades			Percent species discrimination
	*T. glandulifera*	*T. grandiflora*	*Triplostegia* sp. A	
*ndhF*	83	n.d.	n.d.	33.3
*ndhF-trnN*	n.d.	100	n.d.	33.3
*rpoB-trnC*	100	87	98	100
*ycf1*	100	80	90	100
*rbcL*	87	n.d.	n.d.	33.3
*matK*	n.d.	n.d.	n.d.	0
ITS	n.d.	100	80	66.7
*ndhF* + *ndhF-trnN*	71	100	n.d.	66.7
*ndhF* + *rpoB-trnC*	100	95	98	100
*ndhF* + ITS	72	100	94	100
*ndhF-trnN* + *rpoB-trnC*	100	95	98	100
*ndhF-trnN* + ITS	n.d.	100	85	66.7
*rpoB-trnC* + ITS	100	100	100	100
*rbcL + matK*	n.d.	n.d.	n.d.	0
*rbcL + ITS*	n.d.	100	84	66.7
*matK + ITS*	n.d.	100	93	66.7
*ndhF* + *ndhF-trnN* + *rpoB-trnC*	100	100	92	100
*ndhF* + *ndhF-trnN* + ITS	75	100	89	100
*ndhF* + *rpoB-trnC* + ITS	100	100	100	100
*ndhF-trnN* + *rpoB-trnC* + ITS	100	100	99	100
*ndhF* + *ndhF-trnN* + *rpoB-trnC*+ ITS	100	100	99	100
*rbcL + matK + ITS*	n.d.	100	93	66.7
plastid genome	100	100	100	100

n.d., species failed to form a monophyletic clade with bootstrap value ≥70% and thus assigned “not discriminated, n.d.”. The standard plant DNA barcode *trnH-psbA* was not included in our tree-based analyses due to an insufficient number of informative sites for species discrimination

### Morphological and functional traits

**Fig. 7:**
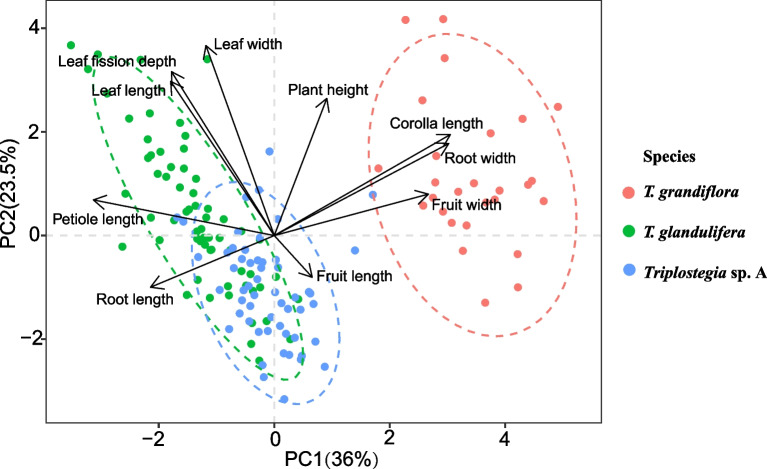
Principal component analysis (PCA) of 10 morphological traits of the three *Triplostegia* species. Morphological trait data were collected by measuring specimens of *Triplostegia*

Except for fruit length, all of the measured morphological traits exhibited significant differences among the three *Triplostegia* species (Table 4). *Triplostegia* sp. A was the shortest plant (28.61 ± 16.28 cm), while *T. grandiflora* was the tallest (44.01 ± 12.56 cm). The taproot of *T. grandiflora* was significantly shorter but thicker compared to that of its two congeners. *Triplostegia* sp. A had serrated leaves, whereas *T. glandulifera* and *T. grandiflora* had pinnatifid and pinnatilobate leaves, respectively. The petiole was 26.55 ± 9.88 mm in *T. glandulifera*, 14.77 ± 5.2 mm in *Triplostegia* sp. A, and absent in *T. grandiflora*. In addition, the corolla of *T. grandiflora* (6.61 ± 1.11 mm) was approximately three times longer than that of *T. glandulifera* (2.31 ± 0.52 mm) and *Triplostegia* sp. A (2.24 ± 0.77 mm) (Table 4). The PCA analysis showed that the 10 morphological traits clearly distinguished *T. grandiflora* from its two congeners, but the other two taxa formed overlapping clusters (Fig. 7; Table S8). Ecologically, *Triplostegia* sp. A typically occurred at higher elevations (3229 ± 382 m) compared to *T. glandulifera* (2284 ± 555 m) and *T. grandiflora* (2450 ± 548 m) (Table 4).

**Table 4:** Differences in elevation and morphological traits difference among the three *Triplostegia* species

Elevation and Traits	*T. glandulifera*	*T. grandiflora*	*Triplostegia* sp. A	*P* value
	Mean ± SD	Mean ± SD	Mean ± SD	
Elevation	2284 ± 555 b	2450 ± 548 b	3229 ± 382 a	**< 0.001**
Plant height (cm)	32.85 ± 11.2 b	44.01 ± 12.56 a	28.61 ± 16.28 c	**< 0.001**
Taproot length (mm)	75.28 ± 32.1 a	41.74 ± 7.78 b	81.29 ± 28.22 a	**< 0.001**
Taproot width (mm)	2.1 ± 0.7 b	6.91 ± 1.74 a	2.49 ± 0.78 b	**< 0.001**
Leaf length (mm)	63.74 ± 24.26 a	49.04 ± 19.89 b	55.84 ± 15.61 a	**0.004**
Leaf width (mm)	23.84 ± 5.87 a	21.58 ± 5.97 ab	19.69 ± 4.83 ab	**< 0.001**
Petiole length (mm)	26.55 ± 9.88 a	0 c	14.77 ± 5.2 b	**< 0.001**
Leaf fission depth (mm)	9.35 ± 3.09 a	5.57 ± 2.02 b	3.81 ± 1.68 c	**< 0.001**
Corolla length (mm)	2.31 ± 0.52 b	6.61 ± 1.11 a	2.24 ± 0.77 b	**< 0.001**
Fruit length (mm)	2.84 ± 0.4	2.91 ± 0.69	3 ± 0.71	0.426
Fruit width (mm)	1.15 ± 0.18 b	1.61 ± 0.26 a	1.28 ± 0.41 b	**< 0.001**

Seven of the eight morphological and functional traits measured in the field showed significant differences between *T. grandiflora* and *Triplostegia* sp. A, the exception being leaf dry mass (Fig. S8-9; Table S9-10). The plants of *T. grandiflora* usually inhabited lower elevations (mean elevation ± SD: 2787 ± 312 m) with a narrower elevation range (2066–3128 m) compared to *Triplostegia* sp. A (3073 ± 306 m; elevation range: 2651–3954 m). The plant height of *T. grandiflora* (height: 36 ± 12 cm) was significantly greater than that of *Triplostegia* sp. (32 ± 13 cm), although *Triplostegia* sp. A exhibiting significant variation in plant height in northwestern Yunnan Province. Furthermore, the leaves of *T. grandiflora* were significantly smaller (mean leaf area of 4.78 ± 2.31 cm^2^) but thicker compared to *Triplostegia* sp. A (8.74 ± 5.47 cm^2^). *Triplostegia grandiflora* had a higher leaf chlorophyll content (47.70 ± 5.93) compared to *Triplostegia* sp. (29.83 ± 5.67). The SLA of *T. grandiflora* (169.72 ± 34.85 cm^2^ / g) was significantly smaller than that of *Triplostegia* sp. A (281.96 ± 72.49 cm^2^ / g). Furthermore, the mean corolla length of *T. grandiflora* (6.19 ± 1.52 mm) was about 3.9 times longer than that of *Triplostegia* sp. A (1.57 ± 0.25 mm) (Fig. S9; Table S9).

### Species distribution modeling

The AUC values for all models in this study were > 0.99, indicating high model performance (Table S11). Precipitation of the warmest quarter (Bio18) was the most important bioclimatic variable in determining the geographical distribution of all three *Triplostegia* species, with a particularly strong influence on *T. glandulifera*. The mean temperature of the coldest quarter (Bio11) was the second most important bioclimatic variable for *T. glandulifera*, while both the mean temperature of the coldest quarter (Bio11) and isothermality (Bio3) were the next most important variables for both *T. grandiflora* and *Triplostegia* sp. A (Fig. S10; Table S12–14).

The potential suitable habitats for *Triplostegia* sp. A exhibited similarities with those of *T. grandiflora* during each time period, with the current predicted range largely confined to the HDM and the Himalaya. However, the potential suitable habitat for *T. glandulifera* was larger, extending to East Asia (Fig. S11). The projected past suitable habitats for each *Triplostegia* species were much smaller than their current suitable habitats, especially for *T. glandulifera* during LIG and LGM. Moreover, all three *Triplostegia* species are projected to experience pronounced habitat shrinkage by 2090. Under the moderate (SSP2–4.5) and pessimistic (SSP5–8.5) climate change scenarios, the suitable areas for *T. glandulifera* are estimated to decrease by 19.4 × 10^4^ km^2^ (19.2%) and 17.54 × 10^4^ km^2^ (17.36%), respectively; *T. grandiflora* will decrease by 9.13× 10^4^km^2^ (19.7%) and 20.79 × 10^4^ km^2^ (44.89%); and *Triplostegia* sp. will decrease by 6.49× 10^4^km^2^ (13.94%) and 9.37 × 10^4^ km^2^ (20.13%), respectively (Fig. S12; Table S15).

**Fig. 8:**
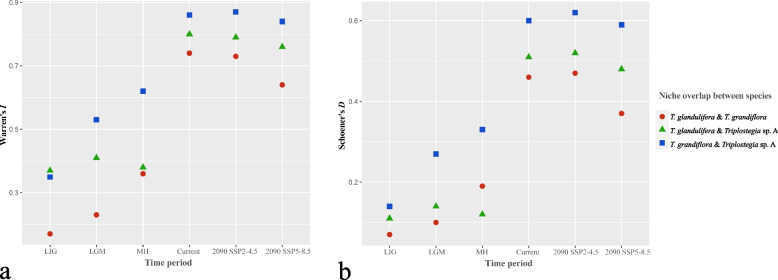
Niche overlap between *Triplostegia* species measured using Warren’s *I* (**a**) and Schoener’s *D* (**b**) indices in different time periods and future climate change scenarios, including Last Interglacial (LIG), Last Glacial Maximum (LGM), mid Holocene (MH), current, and 2090 under SSP2–4.5 and SSP5–8.5 scenarios

The niche overlap between *T. grandiflora* and *Triplostegia* sp. was the largest, followed by that between *T. glandulifera* and *Triplostegia* sp., while *T. grandiflora* and *glandulifera* showed the smallest niche overlap (Fig. 8; Table S16).

## Discussion

### *Confirmation of a third species in* Triplostegia

Our phylogenetic analyses based on datasets of complete plastid genomes (Fig. 3), plastid CDS (Fig. S5), and highly variable plastid DNA regions, consistently indicate that *Triplostegia* contains three well-supported monophyletic species: *T. glandulifera* (BS_ML_ = 100%, PP_BI_ = 1.00 from complete plastid genome data), *T. grandiflora* (BS_ML_ = 100%, PP_BI_ = 1.00), and an undescribed species *Triplostegia* sp. A (BS_ML_ = 100%, PP_BI_ = 1.00). Of these, *T. glandulifera* branched off first, making the other two sister species (Fig. 3). This topology is actually consistent with a previous study [59], which examined fewer accessions and did not distinguish *Triplostegia* sp. A (their accession numbers of SRS3196660, SRS3196661, and SRS3196663) from *T. grandiflora*. Hence, molecular phylogenetic analyses may produce accurate topologies but alone cannot with certainly detect cryptic species. Our Neighbor-net analysis of combined plastid and nuclear data likewise indicated the division of *Triplostegia* into three distinct clusters (Fig. 4). Nuclear data alone did not conflict the plastome-based topology, but samples of *T. glandulifera* did not form a well-supported monophyletic clade (Fig. S6), most likely due to limited resolution from the small part of the genome sampled.

*Triplostegia* sp. A is clearly defined by morphology, as well as plastid data. It differs from its closer relative *T. grandiflora* in seven morphological traits (Table 4), whereas three traits (plant height, petiole length and degree of leaf division) provide consistent differences between all species (Table 4). Ecologically, there is a clear separation by altitude, with sp. A occurring from 1800 to 4342 m, compared to 1800–3200 m for *T. grandiflora* and 1250–3400 m for *T. glandulifera*. The wider altitude range of the high altitude sp. A supports the Rapoport’s Rule [103], which postulates that species at higher elevations tend to have larger elevation ranges. The differing altitude ranges might also contribute to the differing geographical ranges of the three species (Fig. 1). Although *T. grandiflora* and *T. glandulifera* are the most similar pair for altitude range, our ecological niche modeling results indicated that the greatest interspecific niche overlap was between *T. grandiflora* and sp. A (Fig. 8), indicating a correlation between niche overlap and relatedness, and hence phylogenetic conservatism [104]. Hence niche differentiation likely played a significant role in the species diversification of *Triplostegia*.

Functional trait differences between sp. A and *T. grandiflora* appear to be consistent with their ecological separation: a higher chlorophyll content in *T. grandiflora* indicates greater photosynthetic capacity [105], whereas its lower SLA (Fig. S9; Table S9) would normally indicate a resource-stressed environment [88]. Its leaves are also sessile and smaller but thicker, and it has a thicker taproot for water and nutrient storage (Table 4, Fig. S8-9; Table S9), consistent with it occupying a warmer and drier habitat.

Although sp. A. occasionally coexists with *T. grandiflora* in Yunnan where their altitude ranges overlap, we found no morphological intermediates nor other evidence of hybridization. Therefore, they are able to maintain distinct populations even where sympatric. Therefore, based on an integrative examination of molecular, morphological and ecological data, it is clear that sp. A represents an undescribed third species within the genus.

The phylogenetic position of *Triplostegia* has long been controversial [106, 107], but our phylogenetic analysis of Dipsacales based on plastid genomes provides compelling evidence that *Triplostegia* is a monophyletic genus, sister to a clade comprising *Dipsacus*, *Scabiosa*, and *Pterocephalus* (Fig. 2). This is consistent with previous phylogenetic reconstructions of Dipsacales based on plastid genomes, which sampled fewer *Triplostegia* individuals [53, 55].

### Geography, climate and causes of speciation

Recent rapid speciation is a feature of the Hengduan Mountains Region (HDM) [48, 49], thought to be driven by the uplift of the Hengduan Mountains and the late Miocene to Pliocene intensification of the Asian monsoon [46–48, 108, 109]. Such rapid uplifts create new niches at high altitude which newly formed species may inhabit, e.g. the homoploid hybrid species *Pinus densata* [110, 111]. *Triplostegia* sp. A and *T. grandiflora* represent a high/low altitude species pair in the HDM region, similar to *Roscoea humeana* and *R. cautleoides* [112]*.* The altitude ranges of *T. glandulifera* and *T. grandiflora* are fairly similar (Table 4), which indicates that the most recent common ancestor (MRCA) of the genus, and also the sp. A-*T. grandiflora* species pair, probably occupied lower altitudes. If so, the speciation event that produced sp. A might have involved an incursion into colder and/or higher altitude conditions. The timing of this split, around 1 million years ago, indicates that it might have come about due to Quaternary climate fluctuations, with one lineage adapting to cooling conditions coming out of an interglacial while the other moved to lower altitudes or latitudes, tracking the climate.

Large rivers may act as barriers to gene flow [113–116], with examples within China for animals [117, 118], fungi [119], and plants [120, 121]. However, both *T. grandiflora* and *Triplostegia* sp. A occur on both sides of the steep-sided Jinsha River gorge (Fig. 6), indicating that this river gorge is easily traversed, as found for *Roscoea* [112]*.* We observed that the glandular pubescent fruit of *Triplostegia* [52] can easily attach to animal fur and human clothing, potentially facilitating dispersal across the river barrier. River gorges serve as strong barriers for *Vitex negundo* [120] and *Parrotia subaequalis* [121], both of which have seeds apparently dispersed by gravity, whereas certain species in the Amazon region of South America are not affected by river barriers to dispersal [116]. These results suggest that the barrier effect of river gorges largely depends on the specific dispersal traits of plants [115]. A more comprehensive analysis of dispersal and gene flow in *Triplostegia* will require the sampling of more than one individual per population, however.

### Plastid genome features and nucleotide diversity

Newly originated species typically have a narrow geographical range and lower levels of genetic diversity compared to more ancient and widespread congeners [122]. Consistent with this, *T. glandulifera* diverged ~ 7.94 Ma, has the widest distribution range (Fig. 1), and according to Neighbour-Net analysis (Fig. 4) and nucleotide diversity (π) exhibits much higher levels of intraspecific genetic variation than that the other two species, which diverged from each other ~ 1.05 Ma. Likewise plastid genome size varied by 1215 bp within *T. glandulifera* (Table 1, Fig. S1), due to expansion and contraction of the IR/SC boundary regions (Fig. S4), which is a major mechanism underlying plastid genome size variation in plants [28, 39, 123]. Length variation within *T. grandiflora* was 68 bp, and it had the least genetic variation (π) in general consistent with this species having the smallest geographical range of the three (Fig. 1), whereas *Triplostegia* sp. A was intermediate for both range and genetic variation. Otherwise, *Triplostegia* had a high level of conservation in plastid genome structure, gene order, gene content, and genome size, consistent with previous work on the genus [59] and family (Caprifoliaceae) [54, 55, 124–126].

### DNA barcodes for species discrimination

The standard plant DNA barcodes, including *rbcL*, *matK*, *trnH-psbA*, and ITS [23], have been widely used in fields such as community ecology [78, 127], invasive species management [128], and forensic identification [129, 130]. But they are not always effective, especially for taxa that have recently diverged or possess complex evolutionary history [26, 27], and none these, either singly or in combinations, were able to discriminate all three *Triplostegia* species. However, the complete plastid genomes and the highly variable cpDNA region *rpoB-trnC* alone successfully discriminated all three *Triplostegia* species, respectively with high bootstrap values (Table 3; Table S7). The *rpoB-trnC* locus is highly variable in the plastid genomes of other plant lineages, such as *Papaver* [131], *Dioscorea* [132], and *Debregeasia* [133]. In addition, the *ycf1* gene, which is highly variable in flowering plants [77], contained species-specific insertions of 66 bp for *T. grandiflora* and 18 bp for *T. glandulifera*, making it a powerful DNA barcode that could discriminate all three *Triplostegia* species (Table 3; Table S7). The specific function of the *ycf1* gene remains to be explored [134, 135], but it or other plastome variation could be linked to the differences in leaf chlorophyll content between *T. grandiflora* and *Triplostegia* sp. A, and other divergences in photosynthesis-related functions. Therefore, either *rpoB-trnC* or *ycf1* can be used as a taxon-specific DNA barcode for discriminating *Triplostegia* species. Our results highlight the potential of developing taxon-specific barcodes for recently diverged taxa based on plastid genome data, which has been successfully applied in many other plant taxa [42, 136].

### Supplementary Information

**Additional file 1.**

## Data Availability

All newly sequenced and annotated plastid genomes generated in this study have been submitted to NCBI (https://www.ncbi.nlm.nih.gov/) with accession numbers from OP554470 to OP583920 listed in Supplementary Table S1. All plastid genome assembly and annotation data are publicly available (https://www.ncbi.nlm.nih.gov/). The online resources of genomic data were downloaded from NCBI (https://www.ncbi.nlm.nih.gov/) accession numbers listed in Supplementary Tables S1 and S2.

## Abbreviations

IR: Inverted repeat
LSC: Long single-copy
SSC: Short single-copy
ML: Maximum-likelihood
CDS: Coding sequences
Ma: Million years ago

## References

[1] Agapow PM, Bininda-Emonds ORP, Crandall KA, Gittleman JL, Mace GM, Marshall JC, Purvis A (2004). The impact of species concept on biodiversity studies. Q Rev Biol.

[2] Carstens BC, Pelletier TA, Reid NM, Satler JD (2013). How to fail at species delimitation. Mol Ecol.

[3] Cazzolla Gatti R, Reich PB, Gamarra JGP, Crowther T, Hui C, Morera A, et al. The number of tree species on earth. Proc Natl Acad Sci U S A. 2022;119(6):e2115329119. 10.1073/pnas.2115329119 PMC8833151 35101981

[4] Bickford D, Lohman DJ, Sodhi NS, Ng PKL, Meier R, Winker K, Ingram KK, Das I (2007). Cryptic species as a window on diversity and conservation. Trends Ecol Evol.

[5] Struck TH, Feder JL, Bendiksby M, Birkeland S, Cerca J, Gusarov VI, Kistenich S, Larsson KH, Liow LH, Nowak MD (2018). Finding evolutionary processes hidden in cryptic species. Trends Ecol Evol.

[6] Renner M (2020). Opportunities and challenges presented by cryptic bryophyte species. Telopea..

[7] Bhunjun CS, Niskanen T, Suwannarach N, Wannathes N, Chen Y-J, McKenzie EHC, Maharachchikumbura SSN, Buyck B, Zhao C-L, Fan Y-G (2022). The numbers of fungi: are the most speciose genera truly diverse?. Fungal Divers.

[8] Li X, Wiens JJ (2023). Estimating global biodiversity: the role of cryptic insect species. Syst Biol.

[9] Liu R, Wang H, Yang JB, Corlett RT, Randle CP, Li DZ, Yu WB (2022). Cryptic species diversification of the *Pedicularis siphonantha* complex (Orobanchaceae) in the mountains of Southwest China since the Pliocene. Front Plant Sci.

[10] Fiser C, Robinson CT, Malard F (2018). Cryptic species as a window into the paradigm shift of the species concept. Mol Ecol.

[11] Carter BE (2012). Species delimitation and cryptic diversity in the moss genus *Scleropodium* (Brachytheciaceae). Mol Phylogenet Evol.

[12] Wu W, Ng WL, Yang JX, Li WM, Ge XJ (2018). High cryptic species diversity is revealed by genome-wide polymorphisms in a wild relative of banana, *Musa itinerans*, and implications for its conservation in subtropical China. BMC Plant Biol.

[13] Liu Y-Y, Jin W-T, Wei X-X, Wang X-Q (2019). Cryptic speciation in the Chinese white pine (*Pinus armandii*): implications for the high species diversity of conifers in the Hengduan Mountains, a global biodiversity hotspot. Mol Phylogenet Evol.

[14] Stern DL (2013). The genetic causes of convergent evolution. Nat Rev Genet.

[15] Yeaman S, Hodgins KA, Lotterhos KE, Suren H, Nadeau S, Degner JC, Nurkowski KA, Smets P, Wang T, Gray LK (2016). Convergent local adaptation to climate in distantly related conifers. Science.

[16] Kinosian SP, Pearse WD, Wolf PG (2020). Cryptic diversity in the model fern genus *Ceratopteris* (Pteridaceae). Mol Phylogenet Evol.

[17] Criado Ruiz D, Villa Machio I, Herrero Nieto A, Nieto FG (2021). Hybridization and cryptic speciation in the Iberian endemic plant genus *Phalacrocarpum* (Asteraceae-anthemideae). Mol Phylogenet Evol.

[18] Soltis DE, Buggs RJA, Doyle JJ, Soltis PS (2010). What we still don't know about polyploidy. Taxon.

[19] Gu YF, Shu JP, Lu YJ, Shen H, Shao W, Zhou Y, Sun QM, Chen JB, Liu BD, Yan YH (2023). Insights into cryptic speciation of quillworts in China. Plant Divers.

[20] Hebert PDN, Cywinska A, Ball SL, DeWaard JR (2003). Biological identifications through DNA barcodes. Proc R Soc B-Biol Sci.

[21] Kress WJ, Wurdack KJ, Zimmer EA, Weigt LA, Janzen DH (2005). Use of DNA barcodes to identify flowering plants. Proc Natl Acad Sci U S A.

[22] Hollingsworth PM, Forrest LL, Spouge JL, Hajibabaei M, Ratnasingham S, van der Bank M, Chase MW, Cowan RS, Erickson DL, Fazekas AJ (2009). A DNA barcode for land plants. Proc Natl Acad Sci U S A.

[23] Li D-Z, Gao L-M, Li H-T, Wang H, Ge X-J, Liu J-Q, Chen Z-D, Zhou S-L, Chen S-L, Yang J-B (2011). Comparative analysis of a large dataset indicates that internal transcribed spacer (ITS) should be incorporated into the core barcode for seed plants. Proc Natl Acad Sci U S A.

[24] Hebert PDN, Penton EH, Burns JM, Janzen DH, Hallwachs W (2004). Ten species in one: DNA barcoding reveals cryptic species in the neotropical skipper butterfly *Astraptes fulgerator*. Proc Natl Acad Sci U S A.

[25] Liu J, Moeller M, Gao L-M, Zhang D-Q, Li D-Z (2011). DNA barcoding for the discrimination of Eurasian yews (*Taxus* L., Taxaceae) and the discovery of cryptic species. Mol Ecol Resour.

[26] Percy DM, Argus GW, Cronk QC, Fazekas AJ, Kesanakurti PR, Burgess KS, Husband BC, Newmaster SG, Barrett SCH, Graham SW (2014). Understanding the spectacular failure of DNA barcoding in willows (*Salix*): does this result from a trans-specific selective sweep?. Mol Ecol.

[27] Yan L-J, Liu J, Moeller M, Zhang L, Zhang X-M, Li D-Z, Gao L-M (2015). DNA barcoding of *Rhododendron* (Ericaceae), the largest Chinese plant genus in biodiversity hotspots of the Himalaya-Hengduan Mountains. Mol Ecol Resour.

[28] Zhu A, Guo W, Gupta S, Fan W, Mower JP (2016). Evolutionary dynamics of the plastid inverted repeat: the effects of expansion, contraction, and loss on substitution rates. New Phytol.

[29] Wicke S, Schneeweiss GM, dePamphilis CW, Muller KF, Quandt D (2011). The evolution of the plastid chromosome in land plants: gene content, gene order, gene function. Plant Mol Biol.

[30] Twyford AD, Ness RW (2017). Strategies for complete plastid genome sequencing. Mol Ecol Resour.

[31] Greiner S, Sobanski J, Bock R (2015). Why are most organelle genomes transmitted maternally?. Bioessays.

[32] Tonti-Filippini J, Nevill PG, Dixon K, Small I (2017). What can we do with 1000 plastid genomes?. Plant J.

[33] Yu X, Yang D, Guo C, Gao L (2018). Plant phylogenomics based on genome-partitioning strategies: Progress and prospects. Plant Divers.

[34] Gitzendanner MA, Soltis PS, Wong GK, Ruhfel BR, Soltis DE (2018). Plastid phylogenomic analysis of green plants: a billion years of evolutionary history. Am J Bot.

[35] Givnish TJ, Zuluaga A, Spalink D, Soto Gomez M, Lam VKY, Saarela JM, Sass C, Iles WJD, de Sousa DJL, Leebens-Mack J (2018). Monocot plastid phylogenomics, timeline, net rates of species diversification, the power of multi-gene analyses, and a functional model for the origin of monocots. Am J Bot.

[36] Li HT, Luo Y, Gan L, Ma PF, Gao LM, Yang JB, Cai J, Gitzendanner MA, Fritsch PW, Zhang T (2021). Plastid phylogenomic insights into relationships of all flowering plant families. BMC Biol.

[37] Nock CJ, Waters DLE, Edwards MA, Bowen SG, Rice N, Cordeiro GM, Henry RJ (2011). Chloroplast genome sequences from total DNA for plant identification. Plant Biotechnol J.

[38] Kane N, Sveinsson S, Dempewolf H, Yang JY, Zhang D, Engels JMM, Cronk Q (2012). Ultra-barcoding in cacao (*Theobroma* spp.; Malvaceae) using whole chloroplast genomes and nuclear ribosomal DNA. Am J Bot.

[39] Yang J-B, Tang M, Li H-T, Zhang Z-R, Li D-Z (2013). Complete chloroplast genome of the genus *Cymbidium*: lights into the species identification, phylogenetic implications and population genetic analyses. BMC Evol Biol.

[40] Fu C-N, Mo Z-Q, Yang J-B, Cai J, Ye L-J, Zou J-Y, Qin H-T, Zheng W, Hollingsworth PM, Li D-Z (2022). Testing genome skimming for species discrimination in the large and taxonomically difficult genus *Rhododendron*. Mol Ecol Resour.

[41] Ji Y, Liu C, Yang J, Jin L, Yang Z, Yang JB (2020). Ultra-barcoding discovers a cryptic species in *Paris yunnanensis* (Melanthiaceae), a medicinally important plant. Front Plant Sci.

[42] Wang J, Fu C-N, Mo Z-Q, Möller M, Yang J-B, Zhang Z-R, Li D-Z, Gao L-M (2022). Testing the complete Plastome for species discrimination, cryptic species discovery and phylogenetic resolution in *Cephalotaxus* (Cephalotaxaceae). Front Plant Sci.

[43] Myers N, Mittermeier RA, Mittermeier CG, da Fonseca GAB, Kent J (2000). Biodiversity hotspots for conservation priorities. Nature.

[44] Mittermeier RA, Turner WR, Larsen FW, Brooks TM, Gascon C, Zachos FE, Habel JC (2011). Global biodiversity conservation: the critical role of hotspots. Biodiversity hotspots: distribution and protection of conservation priority areas.

[45] Li X-H, Zhu X-X, Niu Y, Sun H (2014). Phylogenetic clustering and overdispersion for alpine plants along elevational gradient in the Hengduan Mountains region, Southwest China. J Syst Evol.

[46] Hughes CE, Atchison GW (2015). The ubiquity of alpine plant radiations: from the Andes to the Hengduan Mountains. New Phytol.

[47] Sun H, Zhang J, Deng T, Boufford DE (2017). Origins and evolution of plant diversity in the Hengduan Mountains, China. Plant Divers.

[48] Xing Y, Ree RH (2017). Uplift-driven diversification in the Hengduan Mountains, a temperate biodiversity hotspot. Proc Natl Acad Sci U S A.

[49] Ding W-N, Ree RH, Spicer RA, Xing Y-W (2020). Ancient orogenic and monsoon-driven assembly of the world's richest temperate alpine flora. Science.

[50] Liu J, Moeller M, Provan J, Gao L-M, Poudel RC, Li D-Z (2013). Geological and ecological factors drive cryptic speciation of yews in a biodiversity hotspot. New Phytol.

[51] Mu Q-Y, Yu C-C, Wang Y, Han T-S, Wang H, Ding W-N, Zhang Q-Y, Low SL, Zheng Q-J, Peng C (2022). Comparative phylogeography of *Acanthocalyx* (Caprifoliaceae) reveals distinct genetic structures in the Himalaya–Hengduan Mountains. Alp Bot.

[52] Hong D, Ma L, Barrie F (2011). Dipsacaceae. Flora China.

[53] Xiang C-L, Dong H-J, Landrein S, Zhao F, Yu W-B, Soltis DE, Soltis PS, Backlund A, Wang H-F, Li D-Z (2020). Revisiting the phylogeny of Dipsacales: new insights from phylogenomic analyses of complete plastomic sequences. J Syst Evol.

[54] Wang HX, Liu H, Moore MJ, Landrein S, Liu B, Zhu ZX, Wang HF (2020). Plastid phylogenomic insights into the evolution of the Caprifoliaceae *s.l.* (Dipsacales). Mol Phylogenet Evol.

[55] Park S, Jun M, Park S, Park S (2021). Lineage-specific variation in IR boundary shift events, inversions, and substitution rates among Caprifoliaceae *s.l.* (Dipsacales) Plastomes. Int J Mol Sci.

[56] Zhang WH, Chen ZD, Li JH, Chen HB, Tang YC (2003). Phylogeny of the Dipsacales *s.l.* based on chloroplast *trnL-F* and *ndhF* sequences. Mol Phylogenet Evol.

[57] Pyck N, Smets E (2004). On the systematic position of *Triplostegia* (Dipsacales): a combined molecular and morphological approach. Belgian J Bot.

[58] Jacobs B, Geuten K, Pyck N, Huysmans S, Jansen S, Smets E (2011). Unraveling the phylogeny of *Heptacodium* and *Zabelia* (Caprifoliaceae): an interdisciplinary approach. Syst Bot.

[59] Niu YT, Jabbour F, Barrett RL, Ye JF, Zhang ZZ, Lu KQ, Lu LM, Chen ZD (2018). Combining complete chloroplast genome sequences with target loci data and morphology to resolve species limits in *Triplostegia* (Caprifoliaceae). Mol Phylogenet Evol.

[60] Niu Y-T, Barrett RL, Zhang Z-Z, Lu L-M, Chen Z-D. Taxonomic revision of *Triplostegia* (Caprifoliaceae: Dipsacales). Phytotaxa. 2019;392(1):19–32.

[61] Doyle JJ, Doyle JL (1987). A rapid DNA isolation procedure for small quantities of fresh leaf tissue. Phytochem Bull.

[62] Brown J, Pirrung M, McCue LA, Wren J (2017). FQC dashboard: integrates FastQC results into a web-based, interactive, and extensible FASTQ quality control tool. Bioinformatics.

[63] Bolger AM, Lohse M, Usadel B (2014). Trimmomatic: a flexible trimmer for Illumina sequence data. Bioinformatics.

[64] Jin J-J, Yu W-B, Yang J-B, Song Y, dePamphilis CW, Yi T-S, Li D-Z (2020). GetOrganelle: a fast and versatile toolkit for accurate de novo assembly of organelle genomes. Genome Biol.

[65] Wick RR, Schultz MB, Zobel J, Holt KE (2015). Bandage: interactive visualization of de novo genome assemblies. Bioinformatics.

[66] Tillich M, Lehwark P, Pellizzer T, Ulbricht-Jones ES, Fischer A, Bock R, Greiner S (2017). GeSeq – versatile and accurate annotation of organelle genomes. Nucleic Acids Res.

[67] Greiner S, Lehwark P, Bock R (2019). OrganellarGenomeDRAW (OGDRAW) version 1.3.1: expanded toolkit for the graphical visualization of organellar genomes. Nucleic Acids Res.

[68] Frazer KA, Pachter L, Poliakov A, Rubin EM, Dubchak I (2004). VISTA: computational tools for comparative genomics. Nucleic Acids Res.

[69] Darling ACE, Mau B, Blattner FR, Perna NT (2004). Mauve: multiple alignment of conserved genomic sequence with rearrangements. Genome Res.

[70] Amiryousefi A, Hyvönen J, Poczai P, Hancock J (2018). IRscope: an online program to visualize the junction sites of chloroplast genomes. Bioinformatics.

[71] Rozas J, Ferrer-Mata A, Sanchez-DelBarrio JC, Guirao-Rico S, Librado P, Ramos-Onsins SE, Sanchez-Gracia A (2017). DnaSP 6: DNA sequence polymorphism analysis of large data sets. Mol Biol Evol.

[72] Katoh K, Standley DM (2013). MAFFT multiple sequence alignment software version 7: improvements in performance and usability. Mol Biol Evol.

[73] Posada D, Crandall KA (1998). MODELTEST: testing the model of DNA substitution. Bioinformatics.

[74] Stamatakis A (2014). RAxML version 8: a tool for phylogenetic analysis and post-analysis of large phylogenies. Bioinformatics.

[75] Ronquist F, Teslenko M, van der Mark P, Ayres DL, Darling A, Hohna S, Larget B, Liu L, Suchard MA, Huelsenbeck JP (2012). MrBayes 3.2: efficient Bayesian phylogenetic inference and model choice across a large model space. Syst Biol.

[76] Huson DH, Bryant D (2006). Application of phylogenetic networks in evolutionary studies. Mol Biol Evol.

[77] Dong W, Xu C, Li C, Sun J, Zuo Y, Shi S, Cheng T, Guo J, Zhou S (2015). *ycf1*, the most promising plastid DNA barcode of land plants. Sci Rep.

[78] Tan S-L, Luo Y-H, Hollingsworth PM, Burgess KS, Xu K, Li D-Z, Gao L-M (2018). DNA barcoding herbaceous and woody plant species at a subalpine forest dynamics plot in Southwest China. Ecol Evol.

[79] Kumar S, Stecher G, Li M, Knyaz C, Tamura K (2018). MEGA X: molecular evolutionary genetics analysis across computing platforms. Mol Biol Evol.

[80] Bouckaert R, Heled J, Kuehnert D, Vaughan T, Wu C-H, Xie D (2014). BEAST 2: a software platform for Bayesian evolutionary analysis. PLoS Comput Biol..

[81] Rambaut A, Drummond AJ, Xie D, Baele G, Suchard MA, Susko E (2018). Posterior summarization in Bayesian Phylogenetics using tracer 1.7. Syst Biol.

[82] Bell CD, Donoghue MJ (2005). Dating the dipsacales: comparing models, genes, and evolutionary implications. Am J Bot.

[83] Wang HF, Landrein S, Dong WP, Nie ZL, Kondo K, Funamoto T, Wen J, Zhou SL (2015). Molecular phylogeny and biogeographic diversification of linnaeoideae (Caprifoliaceae s. L.) disjunctly distributed in Eurasia, North America and Mexico. PLoS One.

[84] Baskin JM, Hidayati SN, Baskin CC, Walck JL, Huang Z-Y, Chien C-T (2006). Evolutionary considerations of the presence of both morphophysiological and physiological seed dormancy in the highly advanced euasterids II order Dipsacales. Seed Sci Res.

[85] Wing SL, Alroy J, Hickey LJ (1995). Plant and mammal diversity in the Paleocene to early Eocene of the Bighorn Basin. Paleogeogr Paleoclimatol Paleoecol.

[86] Moore BR, Donoghue MJ (2007). Correlates of diversification in the plant clade dipsacales: geographic movement and evolutionary innovations. Am Nat.

[87] Manchester SR, Donoghue MJ (1995). Winged fruits of Linnaeeae (Caprifoliaceae) in the tertiary of Western North America: *Diplodipelta* gen. Nov. Int J Plant Sci.

[88] Cornelissen JHC, Lavorel S, Garnier E, Diaz S, Buchmann N, Gurvich DE, Reich PB, ter Steege H, Morgan HD, van der Heijden MGA (2003). A handbook of protocols for standardised and easy measurement of plant functional traits worldwide. Aust J Bot.

[89] Perez-Harguindeguy N, Diaz S, Garnier E, Lavorel S, Poorter H, Jaureguiberry P, Bret-Harte MS, Cornwell WK, Craine JM, Gurvich DE (2013). New handbook for standardised measurement of plant functional traits worldwide. Aust J Bot.

[90] Zou J-Y, Luo Y-H, Burgess KS, Tan S-L, Zheng W, Fu C-N, Xu K, Gao L-M (2021). Joint effect of phylogenetic relatedness and trait selection on the elevational distribution of *Rhododendron* species. J Syst Evol.

[91] R Core Team R (2022). R: a language and environment for statistical computing.

[92] Phillips SJ, Anderson RP, Schapire RE (2006). Maximum entropy modeling of species geographic distributions. Ecol Model.

[93] Qiu L, Jacquemyn H, Burgess KS, Zhang LG, Zhou YD, Yang BY, Tan SL (2023). Contrasting range changes of terrestrial orchids under future climate change in China. Sci Total Environ.

[94] O'Neill BC, Kriegler E, Riahi K, Ebi KL, Hallegatte S, Carter TR, Mathur R, van Vuuren DP (2014). A new scenario framework for climate change research: the concept of shared socioeconomic pathways. Clim Chang.

[95] Lobo JM, Jimenez-Valverde A, Real R (2008). AUC: a misleading measure of the performance of predictive distribution models. Glob Ecol Biogeogr.

[96] Swets JA (1988). Measuring the accuracy of diagnostic systems. Science.

[97] Phillips SJ, Dudik M, Elith J, Graham CH, Lehmann A, Leathwick J, Ferrier S (2009). Sample selection bias and presence-only distribution models: implications for background and pseudo-absence data. Ecol Appl.

[98] Radosavljevic A, Anderson RP (2014). Making better MAXENT models of species distributions: complexity, overfitting and evaluation. J Biogeogr.

[99] Hughes AC (2017). Mapping priorities for conservation in Southeast Asia. Biol Conserv.

[100] Warren DL, Glor RE, Turelli M (2010). ENMTools: a toolbox for comparative studies of environmental niche models. Ecography.

[101] Schoener TW (1968). The Anolis lizards of Bimini: resource partitioning in a complex fauna. Ecology.

[102] Warren DL, Glor RE, Turelli M (2008). Environmental niche equivalency versus conservatism: quantitative approaches to niche evolution. Evolution.

[103] Stevens GC (1992). The elevational gradient in altitudinal range: an extension of Rapoport's latitudinal rule to altitude. Am Nat.

[104] Cooper N, Jetz W, Freckleton RP (2010). Phylogenetic comparative approaches for studying niche conservatism. J Evol Biol.

[105] Croft H, Chen JM, Luo X, Bartlett P, Chen B, Staebler RM (2017). Leaf chlorophyll content as a proxy for leaf photosynthetic capacity. Glob Chang Biol.

[106] Backlund A, Nilsson S (1997). Pollen morphology and the systematic position of *Triplostegia* (Dipsacales). Taxon.

[107] Bell CD (2004). Preliminary phylogeny of Valerianaceae (Dipsacales) inferred from nuclear and chloroplast DNA sequence data. Mol Phylogenet Evol.

[108] Chen Y-S, Deng T, Zhou Z, Sun H (2018). Is the east Asian flora ancient or not?. Natl Sci Rev.

[109] Xia XM, Yang MQ, Li CL, Huang SX, Jin WT, Shen TT, Wang F, Li XH, Yoichi W, Zhang LH (2022). Spatiotemporal evolution of the global species diversity of *Rhododendron*. Mol Biol Evol.

[110] Wang XR, Szmidt AE, Savolainen O (2001). Genetic composition and diploid hybrid speciation of a high mountain pine, *Pinus densata*, native to the Tibetan plateau. Genetics.

[111] Ma F, Zhao C, Milne R, Ji M, Chen L, Liu J (2010). Enhanced drought-tolerance in the homoploid hybrid species *Pinus densata*: implication for its habitat divergence from two progenitors. New Phytol.

[112] Zhao J-L, Gugger PF, Xia Y-M, Li Q-J (2016). Ecological divergence of two closely related *Roscoea* species associated with late Quaternary climate change. J Biogeogr.

[113] Wallace AR (1854). On the monkeys of the Amazon. Ann Mag Nat Hist.

[114] Hayes FE, Sewlal JAN (2004). The Amazon River as a dispersal barrier to passerine birds: effects of river width, habitat and taxonomy. J Biogeogr.

[115] Nazareno AG, Dick CW, Lohmann LG (2017). Wide but not impermeable: testing the riverine barrier hypothesis for an Amazonian plant species. Mol Ecol.

[116] Nazareno AG, Dick CW, Lohmann LG (2019). Tangled banks: a landscape genomic evaluation of Wallace's riverine barrier hypothesis for three Amazon plant species. Mol Ecol.

[117] Li R, Chen W, Tu L, Fu J (2009). Rivers as barriers for high elevation amphibians: a phylogeographic analysis of the alpine stream frog of the Hengduan Mountains. J Zool.

[118] He K, Gutiérrez EE, Heming NM, Koepfli KP, Wan T, He S, Jin W, Liu SY, Jiang XL (2019). Cryptic phylogeographic history sheds light on the generation of species diversity in sky-island mountains. J Biogeogr.

[119] Feng B, Zhao Q, Xu J, Qin J, Yang ZL (2016). Drainage isolation and climate change-driven population expansion shape the genetic structures of *tuber indicum* complex in the Hengduan Mountains region. Sci Rep.

[120] Zhang Z-Y, Zheng X-M, Ge S (2007). Population genetic structure of *Vitex negundo* (Verbenaceae) in three-gorge area of the Yangtze River: the riverine barrier to seed dispersal in plants. Biochem Syst Ecol.

[121] Geng Q, Yao Z, Yang J, He J, Wang D, Wang Z, Liu H (2015). Effect of Yangtze River on population genetic structure of the relict plant *Parrotia subaequalis* in eastern China. Ecol Evol.

[122] Gitzendanner MA, Soltis PS (2000). Patterns of genetic variation in rare and widespread plant congeners. Am J Bot.

[123] Zhang Y, Du L, Liu A, Chen J, Wu L, Hu W, Zhang W, Kim K, Lee SC, Yang TJ (2016). The complete chloroplast genome sequences of five *Epimedium* species: lights into phylogenetic and taxonomic analyses. Front Plant Sci.

[124] Fan WB, Wu Y, Yang J, Shahzad K, Li ZH (2018). Comparative chloroplast genomics of Dipsacales species: insights into sequence variation, adaptive evolution, and phylogenetic relationships. Front Plant Sci.

[125] Liu ML, Fan WB, Wang N, Dong PB, Zhang TT, Yue M, Li ZH (2018). Evolutionary analysis of plastid genomes of seven *Lonicera* L. species: implications for sequence divergence and phylogenetic relationships. Int J Mol Sci.

[126] Wang HX, Moore MJ, Barrett RL, Landrein S, Sakaguchi S, Maki M, Wen J, Wang HF (2020). Plastome phylogenomic insights into the Sino-Japanese biogeography of *Diabelia* (Caprifoliaceae). J Syst Evol.

[127] Kress WJ, Erickson DL, Andrew Jones F, Swenson NG, Perez R, Sanjur O, Bermingham E (2009). Plant DNA barcodes and a community phylogeny of a tropical forest dynamics plot in Panama. Proc Natl Acad Sci U S A.

[128] Xu S-Z, Li Z-Y, Jin X-H (2017). DNA barcoding of invasive plants in China: a resource for identifying invasive plants. Mol Ecol Resour.

[129] Ferri G, Corradini B, Ferrari F, Santunione AL, Palazzoli F, Alu M (2015). Forensic botany II, DNA barcode for land plants: which markers after the international agreement?. Forensic Sci Int Genet.

[130] Liu J, Milne RI, Moller M, Zhu G-F, Ye L-J, Luo Y-H, Yang J-B, Wambulwa MC, Wang C-N, Li D-Z (2018). Integrating a comprehensive DNA barcode reference library with a global map of yews (*Taxus* L.) for forensic identification. Mol Ecol Resour.

[131] Zhou J, Cui Y, Chen X, Li Y, Xu Z, Duan B, Li Y, Song J, Yao H (2018). Complete chloroplast genomes of *Papaver rhoeas* and *Papaver orientale*: molecular structures, comparative analysis, and phylogenetic analysis. Molecules.

[132] Xia W, Zhang B, Xing D, Li Y, Wu W, Xiao Y, Sun J, Dou Y, Tang W, Zhang J (2019). Development of high-resolution DNA barcodes for *Dioscorea* species discrimination and phylogenetic analysis. Ecol Evol.

[133] Wang R-N, Milne RI, Du X-Y, Liu J, Wu Z-Y (2020). Characteristics and mutational hotspots of Plastomes in *Debregeasia* (Urticaceae). Front Genet.

[134] Kikuchi S, Bedard J, Hirano M, Hirabayashi Y, Oishi M, Imai M, Takase M, Ide T, Nakai M (2013). Uncovering the protein Translocon at the chloroplast inner envelope membrane. Science.

[135] de Vries J, Sousa FL, Bölter B, Soll J, Gould SB (2015). YCF1: a green TIC?. Plant Cell.

[136] Slipiko M, Myszczynski K, Buczkowska K, Baczkiewicz A, Szczecinska M, Sawicki J (2020). Molecular delimitation of European leafy liverworts of the genus *Calypogeia* based on plastid super-barcodes. BMC Plant Biol.

