# Supplementary material for: Plastome phylogenomics and morphological traits analyses provide new insights into the phylogenetic position, species delimitation and speciation of Triplostegia (Caprifoliaceae)
Source: BMC Plant Biol. 2023 Dec 15;23:645. doi: 10.1186/s12870-023-04663-4 (PMC10722739; doi:10.1186/s12870-023-04663-4)
Supplement: Supplementary file 1 — Additional file 1. [file 12870_2023_4663_MOESM1_ESM.docx]

***Supplementary Material***

**Plastome phylogenomics and morphological traits analyses provide new insights into the phylogenetic position, species delimitation and speciation of *Triplostegia* (Caprifoliaceae)**

Running title: Plastome phylogenomics of *Triplostegia* (Caprifoliaceae)

Qing-Li Fu^1^✝, Zhi-Qiong Mo^2^✝, Xiao-Guo Xiang^1, 3^, Richard I. Milne^4^, Hans Jacquemyn^5^, Kevin S. Burgess^6^, Ya-Nan Sun^1^, Hua Yan^1^^, 3^, Li Qiu^1^, Bo-Yun Yang^1^, Shao-Lin Tan^1^*

^1^ Jiangxi Province Key Laboratory of Plant Resources, School of Life Sciences, Nanchang University, Nanchang, Jiangxi, 330031, China

^2^ CAS Key Laboratory for Plant Diversity and Biogeography of East Asia, Kunming Institute of Botany, Chinese Academy of Sciences, Kunming, Yunnan, 650201, China

^3^ Jiangxi Province Key Laboratory of Watershed Ecosystem Change and Biodiversity, Institute of Life Science, Nanchang University, Nanchang, Jiangxi, 330031, China

^4^ Institute of Molecular Plant Sciences, School of Biological Sciences, University of Edinburgh, Edinburgh EH9 3JH, UK

^5^ KU Leuven, Department of Biology, Plant Conservation and Population Biology, B-3001 Leuven, Belgium

^6^ College of Letters and Sciences, Columbus State University, University System of Georgia, Columbus, GA 31907-5645, USA

***Author for correspondence**: Shao-Lin Tan

E-mail: [tanshaolin@ncu.edu.cn](mailto:tanshaolin@ncu.edu.cn), ORCID: <https://orcid.org/0000-0001-9977-7773>

We compared the IR/SSC and IR/LSC junction regions of the 33 *Triplostegia* plastid genomes (Fig. S4). These junction regions contain seven genes: *rpl2*, *rpl23*, *trnN*, *ndhF*, *ycf1*, *trnI*, and *trnH*. Specifically, the *rpl2* gene was located in the LSC region, approximately 152 bp from the LSC/IRb junction (JLB) in all three species. The length of the *rpl2* gene ranged from 1506 to 1508 bp in *T. glandulifera* and 1505 bp in both *Triplostegia* sp. A and *T. grandiflora*. The *rpl23* gene spanned the JLB, with 134bp in the LSC region and 151bp in the IRb region in all three species. The *trnN* gene was located in the IRb region, approximately 957–1034 bp (*T. glandulifera*), 1104–1109bp (*Triplostegia* sp. A), and 1335 bp (*T. grandiflora*) away from the IRb/SSC junction (JSB) in all three species. The *ndhF* gene was located in the SSC region, positioned around 268–289 bp (*T. glandulifera*), 258 bp (*Triplostegia* sp. A), and 187 bp (*T. grandiflora*) away from the IRb/SSC junction (JSB). The *ycf1* gene spanned the SSC/IRa junction (JSA), with 4734–4753 bp (*T*. *glandulifera*), 4671–4680 bp (*Triplostegia* sp. A), and 4511 bp (*T*. *grandiflora*) in the SSC region, and 173–459 bp (*T*. *glandulifera*), 255–504 bp (*Triplostegia* sp. A), and 685–730 bp (*T*. *grandiflora*) in the IRa region. The *trnN* gene was located in the IRa region, positioned approximately 957–1034 bp (*T*. *glandulifera*), 1104–1109 bp (*Triplostegia* sp. A), and 1335 bp (*T*. *grandiflora*) away from the JSA. The *trnI* gene was located in the IRa region, approximately 316 bp from the LSC/IRa junction (JLA) in all three species. Finally, the *trnH* gene was found in the LSC region, with a distance of 75–84bp away from the JLA for *T*. *glandulifera*, and 69 bp away from the JLA for both *Triplostegia* sp. A and *T*. *grandiflora*.

**Table S1.** Sampling information of the 33 *Triplostegia* plastid genomes analyzed in this study, including 11 recently published plastid genomes*.*

| Species | Locality | Accession No. | Voucher | Longitude(°E) | latitude(°N) | Altitude(m) | References |
| --- | --- | --- | --- | --- | --- | --- | --- |
| *T. glandulifera* | Shennongjia, Hubei | SRS3196653 | Lu289 | 110.0378 | 31.5176 | 1839 | Niu et al. 2018 |
| *T. glandulifera* | Luding, Sichuan | SRS3196654 | Lu165 | 101.9816 | 29.5692 | 3256 | Niu et al. 2018 |
| *T. glandulifera* | Hehuanxibudao, Taiwan | SRS3196655 | Zhong463 | 121.2943 | 24.1544 | 2600 | Niu et al. 2018 |
| *T. glandulifera* | Nantou, Taiwan | SRS3196656 | Chen2011056 | 121.2979 | 24.5424 | 3100 | Niu et al. 2018 |
| *T. glandulifera* | Leibo, Sichuan | SRS3196657 | Chen20110804294 | 103.1794 | 28.2471 | 2500 | Niu et al. 2018 |
| *T. glandulifera* | Wolong, Sichuan | SRS3196658 | WL0142 | 103.2275 | 31.0774 | 2900 | Niu et al. 2018 |
| *T. glandulifera* | Cuona, Xizang | SRS3196663 | Yu34 | 91.8066 | 27.8752 | 2600 | Niu et al. 2018 |
| *T. grandiflora* | Shangri-La, Yunnan | OP554470 | TSL20893 | 100.1190833 | 27.39090833 | 2975 | This study |
| *T. grandiflora* | Shangri-La, Yunnan | OP562874 | TSL201082 | 100.0904511 | 27.6128252 | 3105 | This study |
| *T. grandiflora* | Deqin, Yunnan | OP562875 | TSL201385 | 99.4219864 | 27.8260989 | 3052 | This study |
| *T. grandiflora* | Deqin, Yunnan | OP562876 | TSL201482 | 99.3748208 | 27.7898489 | 2751 | This study |
| *T. grandiflora* | Yulong, Yunnan | OP562877 | TSL201602 | 99.67214722 | 27.160025 | 2990 | This study |
| *T. grandiflora* | Yulong, Yunnan | OP562878 | TSL201740 | 99.83834722 | 26.81063611 | 2102 | This study |
| *T. grandiflora* | Yulong, Yunnan | OP562879 | TSL201772 | 99.75290524 | 26.81161218 | 2856 | This study |
| *T. grandiflora* | Yulong, Yunnan | OP562880 | TSL202291 | 100.1974743 | 26.99848295 | 2713 | This study |
| *T. grandiflora* | Muli, Sichuan | SRS3196659 | Lu102 | 101.0355 | 28.0563 | 2400 | Niu et al. 2018 |
| *Triplostegia* sp. A | Yulong, Yunnan | OP562881 | TSL20213 | 100.347125 | 27.432468 | 3373 | This study |
| *Triplostegia* sp. A | Shangri-La, Yunnan | OP583908 | TSL20552 | 100.0619 | 27.24074167 | 3436 | This study |
| *Triplostegia* sp. A | Shangri-La, Yunnan | OP583909 | TSL20785 | 100.0921889 | 27.39576389 | 3391 | This study |
| *Triplostegia* sp. A | Shangri-La, Yunnan | OP583910 | TSL20922 | 100.0192556 | 27.55766389 | 3392 | This study |
| *Triplostegia* sp. A | Shangri-La, Yunnan | OP583911 | TSL201048 | 100.110559 | 27.6237001 | 3062 | This study |
| *Triplostegia* sp. A | Shangri-La, Yunnan | OP583912 | TSL201190 | 99.620946 | 27.7321188 | 3571 | This study |
| *Triplostegia* sp. A | Deqin, Yunnan | OP583913 | TSL201430 | 99.3435751 | 27.793723 | 3411 | This study |
| *Triplostegia* sp. A | Yulong, Yunnan | OP583914 | TSL201546 | 99.41807778 | 27.43534722 | 3143 | This study |
| *Triplostegia* sp. A | Yulong, Yunnan | OP583915 | TSL201623 | 99.67224167 | 27.16029444 | 3038 | This study |
| *Triplostegia* sp. A | Yulong, Yunnan | OP583916 | TSL201785 | 99.7523988 | 26.81373906 | 2914 | This study |
| *Triplostegia* sp. A | Ninglang, Yunnan | OP583917 | TSL201851 | 100.5500982 | 27.77732832 | 3566 | This study |
| *Triplostegia* sp. A | Ninglang, Yunnan | OP583918 | TSL202113 | 100.712959 | 27.34112669 | 3087 | This study |
| *Triplostegia* sp. A | Ninglang, Yunnan | OP583919 | TSL202158 | 100.5658402 | 27.15988362 | 2773 | This study |
| *Triplostegia* sp. A | Yulong, Yunnan | OP583920 | TSL202293 | 100.1974948 | 27.99814193 | 2737 | This study |
| *T. glandulifera* | Linzhi, Xizang | SRS3196660 | Chen20110804086 | 94.4939 | 29.5765 | 3200 | Niu et al. 2018 |
| *T. glandulifera* | Jilong, Xizang | SRS3196661 | CMTTE937 | 85.305 | 28.859 | 3200 | Niu et al. 2018 |
| *T. glandulifera* | Gesala, Yunnan | SRS3196662 | Lu53 | 101.2487 | 27.1578 | 2876 | Niu et al. 2018 |

**Table S2.** NCBI accession numbers for the 57 plastid genomes used in the phylogenetic reconstruction of Dipsacales in this study*.*

| Families | Species | NCBI accession numbers |
| --- | --- | --- |
| Caprifoliaceae | *Dipsacus asper*Wall. ex DC. | NC039748 |
| Caprifoliaceae | *Dipsacus japonicus*Miq. | MZ934745 |
| Caprifoliaceae | *Scabiosa tschiliensis* Grüning | NC045050 |
| Caprifoliaceae | *Scabiosa comosa*Fisch. ex Roem. & Schult. | NC065840 |
| Caprifoliaceae | *Pterocephalus hookeri* (C.B.Clarke) Airy Shaw & M.L.Green | NC045049 |
| Caprifoliaceae | *Triplostegia glandulifera* Wall. ex DC. SRS3196654 | SRS3196654 |
| Caprifoliaceae | *Triplostegia glandulifera* Wall. ex DC. SRS3196656 | SRS3196656 |
| Caprifoliaceae | *Triplostegia glandulifera* Wall. ex DC. SRS3196657 | SRS3196657 |
| Caprifoliaceae | *Triplostegia grandiflora* Gagnep. TSL201772 | OP562879 |
| Caprifoliaceae | *Triplostegia grandiflora* Gagnep. TSL201602 | OP562877 |
| Caprifoliaceae | *Triplostegia grandiflora* Gagnep. TSL202291 | OP562880 |
| Caprifoliaceae | *Triplostegia* sp. A TSL201190 | OP583912 |
| Caprifoliaceae | *Triplostegia* sp. A TSL20552 | OP583908 |
| Caprifoliaceae | *Triplostegia* sp. A TSL202158 | OP583919 |
| Caprifoliaceae | *Valeriana officinalis*L. | NC045052 |
| Caprifoliaceae | *Valeriana jatamansi* Jones | NC067975 |
| Caprifoliaceae | *Patrinia heterophylla* Bunge | NC045047 |
| Caprifoliaceae | *Patrinia monandra*C. B. Clarke | NC061006 |
| Caprifoliaceae | *Kolkwitzia amabilis* Graebn. | NC029874 |
| Caprifoliaceae | *Diabelia sanguinea* (Makino) Landrein | NC045193 |
| Caprifoliaceae | *Dipelta yunnanensis* Franch. | NC042201 |
| Caprifoliaceae | *Abelia chinensis*R. Br. | NC_045043 |
| Caprifoliaceae | *Linnaea borealis* L. | MN548092 |
| Caprifoliaceae | *Acanthocalyx alba* (Hand.-Mazz.) M.J.Cannon | NC045055 |
| Caprifoliaceae | *Acanthocalyx nepalensis*(D. Don) M. Cannon | MW053690 |
| Caprifoliaceae | *Morina longifolia* Wall. ex DC | NC045046 |
| Caprifoliaceae | *Morina chinensis* Y.Y.Pai | NC069295 |
| Caprifoliaceae | *Zabelia dielsii* (Graebn.) Makino | NC046599 |
| Caprifoliaceae | *Zabelia biflora*(Turcz.) Makino ex Hisauti & H.Hara | NC045063 |
| Caprifoliaceae | *Lonicera ferdinandi* Franch. | NC040963 |
| Caprifoliaceae | *Lonicera japonica*Thunb. | MZ779026 |
| Caprifoliaceae | *Lonicera tangutica*Maxim. | OP388442 |
| Caprifoliaceae | *Lonicera confusa*DC. | NC045045 |
| Caprifoliaceae | *Symphoricarpos sinensis* Rehder | NC054265 |
| Caprifoliaceae | *Leycesteria formosa* Wall. | NC057000 |
| Caprifoliaceae | *Triosteum* *pinnatifidum*Maxim. | NC037952 |
| Caprifoliaceae | *Triosteum himalayanum* Wall. | NC045219 |
| Caprifoliaceae | *Heptacodium miconioides*Rehd. | NC042739 |
| Caprifoliaceae | *Weigela florida* (Bunge) A. DC. | NC037950 |
| Caprifoliaceae | *Diervilla sessilifolia* Buckley | NC064360 |
| Adxoxaceae | *Viburnum dilatatum* Thunb. | NC057163 |
| Adxoxaceae | *Viburnum cylindricum* Buch-Ham. ex D. Don | ON881798 |
| Adxoxaceae | *Viburnum opulus*L. | LT996894 |
| Adxoxaceae | *Viburnum carlesii*Hemsl. | MN985820 |
| Adxoxaceae | *Viburnum utile*Hemsl. | NC032296 |
| Adxoxaceae | *Tetradoxa omeiensis*(H.Hara) C.Y.Wu | NC034793 |
| Adxoxaceae | *Adoxa moschatellina*L. | NC034792 |
| Adxoxaceae | *Sinadoxa corydalifolia*C.Y.Wu, Z.L.Wu & R.F.Huang | NC032040 |
| Adxoxaceae | *Sambucus nigra*L. | NC045061 |
| Adxoxaceae | *Sambucus javanica*Reinw. ex Blume | MT457822 |
| Adxoxaceae | *Sambucus williamsii*Hance | NC033878 |
| Adxoxaceae | *Sambucus adnata*Wall. | MW007718 |
| Paracryphiaceae | *Quintinia verdonii*F.Muell. | NC041281 |
| Apiaceae | *Apium graveolens* L. | NC041087 |
| Pittosporaceae | *Pittosporum kerrii* Craib | NC046847 |
| Pedaliaceae | *Sesamum indicum* L. | NC016433 |
| Lamiaceae | *Mentha spicata* L. | NC037247 |

**Table S3.** NCBI accession numbers of plastid genomes used to estimate the divergence time of Dipsacales.

| Families | Species | NCBI accession numbers |
| --- | --- | --- |
| Caprifoliaceae | *Linnaea borealis* L. | MN548092 |
| Caprifoliaceae | *Kolkwitzia amabilis* Graebn. | NC029874 |
| Caprifoliaceae | *Dipelta yunnanensis* Franch. | NC042201 |
| Caprifoliaceae | *Diabelia* *sanguinea* (Makino) Landrein | NC045193 |
| Caprifoliaceae | *Zabelia dielsii* (Graebn.) Makino | NC046599 |
| Caprifoliaceae | *Dipsacus asper* Wall. ex DC | NC039748 |
| Caprifoliaceae | *Scabiosa tschiliensis* Grüning | NC045050 |
| Caprifoliaceae | *Pterocephalus hookeri* (C. B. Clarke) Airy Shaw & M.L.Green | NC045049 |
| Caprifoliaceae | *Triplostegia glandulifera* Wall. ex DC. | SRS3196654 |
| Caprifoliaceae | *Triplostegia* sp. A | OP583912 |
| Caprifoliaceae | *Triplostegia grandiflora* Gagnep. | OP562877 |
| Caprifoliaceae | *Patrinia heterophylla* Bunge | NC045047 |
| Caprifoliaceae | *Valeriana officinalis*L. | NC045052 |
| Caprifoliaceae | *Morina longifolia* Wall. ex DC. | NC045046 |
| Caprifoliaceae | *Acanthocalyx alba* (Hand.-Mazz.) M.J.Cannon | NC045055 |
| Caprifoliaceae | *Triosteum pinnatifidum* Maxim. | NC037952 |
| Caprifoliaceae | *Symphoricarpos sinensis* Rehder | NC054265 |
| Caprifoliaceae | *Leycesteria formosa* Wall. | NC057000 |
| Caprifoliaceae | *Lonicera ferdinandi* Franch. | NC040963 |
| Caprifoliaceae | *Heptacodium miconioides*Rehder | NC042739 |
| Caprifoliaceae | *Weigela florida* (Bunge) A.DC. | NC037950 |
| Caprifoliaceae | *Diervilla sessilifolia* Buckley | NC064360 |
| Adoxaceae | *Sinadoxa corydalifolia*C.Y.Wu, Z.L.Wu & R.F.Huang | NC032040 |
| Adoxaceae | *Adoxa moschatellina* L. | NC034792 |
| Adoxaceae | *Tetradoxa omeiensis* (H.Hara) C. Y. Wu | NC034793 |
| Adoxaceae | *Sambucus nigra* L. | NC045061 |
| Adoxaceae | *Viburnum dilatatum* Thunb. | NC057163 |
| Apiaceae | *Apium graveolens* L. | NC041087 |
| Pittosporaceae | *Pittosporum kerrii* Craib | NC046847 |
| Pedaliaceae | *Sesamum indicum* L. | NC016433 |
| Lamiaceae | *Mentha spicata* L. | NC037247 |

**Table S4.** Selection of the best-fit nucleotide substitution model using ModelTest v.3.7 based on the Akaike information criterion (AIC).

| Model | -lnL | K | AIC | delta | Weight | CumWeight |
| --- | --- | --- | --- | --- | --- | --- |
| GTR+I+G | 232760.6 | 10 | 465541.2 | 0 | 1 | 1 |
| GTR+G | 232907.7 | 9 | 465833.5 | 292.3125 | 0.00E+00 | 1 |
| GTR+I | 233179.5 | 9 | 466377 | 835.875 | 0.00E+00 | 1 |
| HKY+I+G | 233868.7 | 6 | 467749.4 | 2208.281 | 0.00E+00 | 1 |
| HKY+G | 234022.9 | 5 | 468055.7 | 2514.563 | 0.00E+00 | 1 |
| HKY+I | 234266.5 | 5 | 468543.1 | 3001.906 | 0.00E+00 | 1 |
| SYM+I+G | 234340.3 | 7 | 468694.6 | 3153.406 | 0.00E+00 | 1 |
| SYM+G | 234509.9 | 6 | 469031.8 | 3490.656 | 0.00E+00 | 1 |
| SYM+I | 234761 | 6 | 469534.1 | 3992.906 | 0.00E+00 | 1 |
| K80+I+G | 235068.9 | 3 | 470143.8 | 4602.594 | 0.00E+00 | 1 |
| K80+G | 235242.4 | 2 | 470488.8 | 4947.594 | 0.00E+00 | 1 |
| K80+I | 235490.9 | 2 | 470985.8 | 5444.625 | 0.00E+00 | 1 |
| F81+I+G | 237832.3 | 5 | 475674.6 | 10133.41 | 0.00E+00 | 1 |
| F81+G | 237985.1 | 4 | 475978.1 | 10436.97 | 0.00E+00 | 1 |
| F81+I | 238224.4 | 4 | 476456.8 | 10915.66 | 0.00E+00 | 1 |
| GTR | 238886.1 | 8 | 477788.2 | 12247.03 | 0.00E+00 | 1 |
| JC+I+G | 238898.6 | 2 | 477801.2 | 12260.03 | 0.00E+00 | 1 |
| JC+G | 239061.7 | 1 | 478125.4 | 12584.22 | 0.00E+00 | 1 |
| JC+I | 239305.6 | 1 | 478613.2 | 13072.03 | 0.00E+00 | 1 |
| HKY | 240109.9 | 4 | 480227.8 | 14686.59 | 0.00E+00 | 1 |
| SYM | 240678.9 | 5 | 481367.9 | 15826.72 | 0.00E+00 | 1 |
| K80 | 241500.1 | 1 | 483002.3 | 17461.09 | 0.00E+00 | 1 |
| F81 | 244064.4 | 3 | 488134.8 | 22593.59 | 0.00E+00 | 1 |
| JC | 245236.9 | 0 | 490473.8 | 24932.69 | 0.00E+00 | 1 |

Note: -lnL: negative log likelihood, K: number of estimated (free) parameters, AIC: Akaike Information Criterion, delta: Akaike difference, weight: Akaike weight, cumWeight: cumulative Akaike weight

**Table S5.** The environmental variables used in MaxEnt model.

| Code | Description | Units |
| --- | --- | --- |
| Bio1 | Annual mean temperature | ℃ |
| Bio2 | Mean monthly temperature difference | ℃ |
| Bio3 | Isothermality（Bio2/Bio7）(*100) | – |
| Bio4 | Temperature Seasonality (standard deviation*100) | – |
| Bio5 | Maximum temperature of warmest month | ℃ |
| Bio6 | Minimum temperature of coldest month | ℃ |
| Bio7 | Temperature annual range | ℃ |
| Bio8 | Mean temperature of wettest quarter | ℃ |
| Bio9 | Mean temperature of driest quarter | ℃ |
| Bio10 | Mean temperature of warmest quarter | ℃ |
| Bio11 | Mean temperature of coldest quarter | ℃ |
| Bio12 | Annual precipitation | mm |
| Bio13 | Precipitation of wettest period | mm |
| Bio14 | Precipitation of driest period | mm |
| Bio15 | Precipitation seasonality | – |
| Bio16 | Precipitation of wettest quarter | mm |
| Bio17 | Precipitation of driest quarter | mm |
| Bio18 | Precipitation of warmest quarter | mm |
| Bio19 | Precipitation of coldest quarter | mm |

**Table S6.** Chloroplast genes of *Triplostegia.*

| Category | Gene Groups | Gene Names |
| --- | --- | --- |
| RNA genes | Ribosomal RNA genes (rRNA) | *rrn16*^a^、*rrn23* ^a^、*rrn4.5* ^a^、*rrn5* ^a^ |
|  |  | *trnH-GUG* *trnK-UUU*^+^ *trnQ-UUG* *trnS-GCU* *trnG-GCC* *trnR-UCU* *trnC-GCA* *trnD-GUC* *trnY-GUA* *trnE-UUC* *trnT-GGU* *trnS-UGA* *trnG-UCC* *trnfM-CAU* *trnS-GGA trnT-GUG trnL-UAA*^+^ *trnF-GAA* *trnV-UAC*^+^ *trnM-CAU* *trnW-CCA* *trnP-UGG* *trnI-CAU*^a^ *trnL-CAA*^a^ *trnV-GAC*^a^ *trnI-GAU*^+,a^ *trnA-UGC*^+,a^ *trnR-ACG*^a^ *trnN-GUU*^a^ *trnL-UAG* |
|  |  |  |
|  |  |  |
|  |  |  |
|  | Transfer RNA genes (tRNA) |  |
|  |  |  |
|  |  |  |
|  |  |  |
|  |  |  |
|  |  |  |
| Ribosomal proteins | Small ribosomal subunit | *rps16*^+^ *rps2* *rps14* *rps4* *rps18* *rps12*^+,a^ *rps11* *rps8* *rps3* *rps19* *rps7*^a^ *rps15* |
| Transcription | Large ribosomal subunit | *rpl33* *rpl20* *rpl36 rpl14 rpl16*^+^ *rpl22 rpl2*^+^ *rpl23*^a^ *rpl32* |
|  | DNA dependent RNA polymerase | *rpoC2 rpoC1*^+^ *rpoB rpoA* |
| Protein-coding genes | Photosystem I | *psaB psaA psaI psaJ psaC* |
|  | Photosystem II | *psbA psbK psbI psbM psbD psbC psbZ psbJ psbL psbF psbE psbB psbT psbN psbH* |
|  | Subunit of cytochrome | *petN petA petL petG petB*^+^ *petD*^+^ |
|  | Subunit of synthase | *atpA atpF*^+^ *atpH atpI atpE atpB* |
|  | Large subunit of Rubisco | *rbcL* |
|  | NADH dehydrogenase | *ndhJ ndhK ndhC ndhB^+,a^ ndhF ndhD ndhE ndhG ndhI ndhA^+^ ndhH* |
| Other genes | ATP dependent protease subunit P | *clpP*^++^ |
|  | Chloroplast envelope membrane protein | *cemA* |
|  | Maturase | *matK* |
|  | Subunit of acetyl-CoA carboxylase | *accD* |
|  | C-type cytochrome synthesis | *ccsA* |
|  | Translation initiation factor | *infA* |
|  | Conserved open reading frames | *ycf3*^++^ *ycf4* *ycf2*^+,a^ *ycf1* ^a^ |

Note: +, Gene with one intron; ++, gene with two introns and a gene with multiple copies; a, gene with two copies.

**Table S7.** Species discrimination rates of *Triplostegia* based on highly variable plastid DNA regions and standard plant DNA barcodes using distance-based analysis. Pairwise interspecific and intraspecific genetic distances were calculated based on the Kimura 2-parameter (k2p) mode.

| DNA Regions | *T. glandulifera* | |  | *T. grandiflora* | | |  | *Triplostegia* sp. A | | Percent species  discrimination |
| --- | --- | --- | --- | --- | --- | --- | --- | --- | --- | --- |
|  | intra k2p | inter k2p |  | | intra k2p | inter k2p |  | intra k2p | inter k2p |  |
| *ndhF* | 0-0.0035 | 0.0044-0.0067 |  | | 0 | 0.0004-0.0067 |  | 0 | 0.0004-0.0067 | 100 |
| *ndhF-trnN* | 0-0.0078 | 0.0133-0.0440 |  | | 0-0.0007 | 0.0239-0.0440 |  | 0 | 0.0133-0.0247 | 100 |
| *rpoB-trnC* | 0-0.0063 | 0.0119-0.0151 |  | | 0 | 0.0023-0.0151 |  | 0-0.0008 | 0.0023-0.0151 | 100 |
| *ycf1* | 0-0.0063 | 0.0076-0.0092 |  | | 0-0.0004 | 0.0014-0.0092 |  | 0-0.0004 | 0.0014-0.0086 | 100 |
| *rbcL* | 0-0.0021 | 0.0014-0.0042 |  | | 0 | 0-0.0035 |  | 0-0.0007 | 0-0.0042 | 0 |
| *matK* | 0-0.0033 | 0.0033-0.0060 |  | | 0 | 0-0.0053 |  | 0-0.0007 | 0-0.0060 | 0 |
| *trnH-psbA* | 0-0.0172 | 0-0.0172 |  | | 0 | 0-0.0115 |  | 0-0.0057 | 0-0.0172 | 0 |
| ITS | 0-0.0199 | 0.0132-0.0285 |  | | 0 | 0.0133-0.0285 |  | 0-0.0065 | 0.0132-0.0266 | 66.7 |

Note: intra k2p, intraspecific k2p distance; inter k2p, interspecific k2p distance. A species was considered successfully discriminated if the minimum interspecific k2p distance involving this species was larger than the maximum intraspecific k2p distance of this species.

**Table S8.** Principle component analysis (PCA) of ten morphological traits of three *Triplostegia* species and their contribution to PC1, PC2, PC3, and PC4. These ten traits were measured from specimens.

| Principle component | | PC 1 | PC 2 | PC 3 | PC 4 |
| --- | --- | --- | --- | --- | --- |
| Eigenvalues % of Variance | | 35.95 | 23.52 | 12.51 | 7.62 |
| Contribution of  morphological  traits to  different  components | Plant height | 0.13 | 0.38 | -0.14 | -0.69 |
|  | Corolla length | 0.44 | 0.28 | -0.06 | 0.05 |
|  | Leaf fission depth | -0.25 | 0.45 | 0.04 | 0.22 |
|  | Petiole length | -0.45 | 0.10 | 0.12 | 0.08 |
|  | Leaf length | -0.26 | 0.42 | 0.15 | < 0.01 |
|  | Leaf width | -0.17 | 0.52 | 0.13 | 0.10 |
|  | Root length | -0.31 | -0.14 | 0.15 | -0.67 |
|  | Root width | 0.43 | 0.25 | -0.10 | -0.06 |
|  | Fruit length | 0.09 | -0.11 | 0.81 | -0.05 |
|  | Fruit width | 0.38 | 0.11 | 0.48 | 0.01 |

**Table S9.** Comparison of elevation range and eight functional traits between coexisting *Triplostegia* sp. A and *T. grandiflora* in northwest Yunnan, southwest China. Data obtained from field measurements in 2020.

| Elevation and Traits | *T. grandiflora* | | *Triplostegia sp.* A | | W | P value |
| --- | --- | --- | --- | --- | --- | --- |
|  | Range | Mean ± SD | Range | Mean ± SD |  |  |
| Elevation (m) | 2066-3128 | 2787 ± 312 | 2651-3954 | 3073 ± 306 | 12729 | **<0.001 ***** |
| Plant height (cm) | 14-70 | 36 ± 12 | 6-69 | 32 ± 13 | 7058.5 | **0.002 **** |
| Chlorophyll content | 32.10-66.25 | 47.70 ± 5.93 | 18.20-44.30 | 29.83 ± 5.67 | 282 | **< 0.001 ***** |
| Leaf area (cm^2^) | 1.33-12.92 | 4.78 ± 2.31 | 1.89-46.56 | 8.74 ± 5.47 | 14246 | **< 0.001 ***** |
| Leaf thickness (mm) | 0.195-0.415 | 0.313 ± 0.05 | 0.125-0.300 | 0.179 ± 0.030 | 298 | **< 0.001 ***** |
| Leaf dry mass (g) | 0.007-0.078 | 0.029 ± 0.0137 | 0.005-0.151 | 0.031 ± 0.017 | 10063 | 0.1005 |
| SLA (cm^2^ / g) | 102.46-284.76 | 169.72 ± 34.85 | 173.40-489.09 | 281.96 ± 72.49 | 17005 | **< 0.001 ***** |
| Corolla length (mm) | 3.43-10.70 | 6.19 ± 1.52 | 1.06-2.075 | 1.573 ± 0.252 | 0 | **< 0.001 ***** |
| Corolla width (mm) | 1.94-5.95 | 3.73 ± 0.98 | 0.96-2.65 | 1.614 ± 0.335 | 16.5 | **< 0.001 ***** |

Note: W is the Wilcoxon rank sum test value.

**Table S10.** Principle component analysis (PCA) of eight functional traits of coexisting *T. grandiflora* and *Triplostegia* sp. A in northwestern Yunnan, and their contribution to PC1 and PC2.

| Principle component | | PC 1 | PC 2 |
| --- | --- | --- | --- |
| Eigenvalues % of Variance | | 54.88 | 25.68 |
| Contribution of  morphological  traits to  different  components | Plant height | 0.10 | 0.52 |
|  | Chlorophyll concentration | 0.43 | 0.11 |
|  | Leaf thickness | 0.43 | 0.08 |
|  | Leaf area | -0.27 | 0.53 |
|  | Leaf dry matter content | -0.07 | 0.65 |
|  | Specific leaf area | -0.40 | < 0.01 |
|  | Flower length | 0.45 | 0.06 |
|  | Flower width | 0.44 | 0.06 |

**Table S11.** AUC mean values of simulated distribution for *T. glandulifera*, *T. grandiflora*, and *Triplostegia* sp. A in different time periods and future climate change scenarios.

| Species |  | paleoclimatic | | | Current | 2090s | |
| --- | --- | --- | --- | --- | --- | --- | --- |
|  |  | Last Interglacial | Last Glacial Maximum | Mid Holocene |  | SSP2-4.5 | SSP5-8.5 |
| *T. glandulifera* | AUC | 0.995 | 0.995 | 0.995 | 0.996 | 0.995 | 0.995 |
| *T. grandiflora* | AUC | 0.998 | 0.998 | 0.998 | 0.998 | 0.998 | 0.997 |
| *Triplostegia* sp. A | AUC | 0.996 | 0.996 | 0.996 | 0.996 | 0.996 | 0.996 |

**Table S12.** Contribution (%) of environmental variables to the distribution of *T. glandulifera.*

| Time | Bio18 | Bio11 | Bio15 | Bio10 | Bio2 | Bio3 |
| --- | --- | --- | --- | --- | --- | --- |
| Last Interglacial | 51.6 | 28.1 | 11.4 | 6.2 | 2.2 | 0.4 |
| Last Glacial Maximum | 51 | 28.3 | 11.9 | 6.2 | 1.9 | 0.6 |
| Mid Holocene | 50 | 27 | 12.8 | 6.3 | 3.2 | 0.7 |
| Current | 51.1 | 27.1 | 12.3 | 6.6 | 2.3 | 0.6 |
| 2090s SSPs 2-4.5  2090s SSPs 5-8.5 | 53.1 | 25.9 | 12.1 | 5.3 | 2.5 | 1.2 |
|  | 51.4 | 28 | 11.8 | 5.6 | 2.2 | 1 |

**Table S13.** Contribution (%) of environmental variables to the distribution of *T. grandiflora.*

| Time | Bio18 | Bio11 | Bio3 | Bio15 | Bio14 | Bio2 | Bio5 | Bio7 |
| --- | --- | --- | --- | --- | --- | --- | --- | --- |
| Last Interglacial | 40 | 20.4 | 21 | 8.1 | 5.3 | 3.5 | 1.1 | 0.5 |
| Last Glacial Maximum | 39.5 | 21.7 | 19.1 | 8.4 | 5.9 | 2.9 | 1.7 | 0.8 |
| Mid Holocene | 42.7 | 21.7 | 18.6 | 7.4 | 4.6 | 3.6 | 0.4 | 1.1 |
| Current | 43 | 21.6 | 20.7 | 7.9 | 3.1 | 3.3 | 0.1 | 0.2 |
| 2090s SSPs 2-4.5  2090s SSPs 5-8.5 | 40 | 20.5 | 20 | 8.3 | 5.6 | 3.6 | 1.6 | 0.4 |
|  | 42.5 | 22 | 19.2 | 8.3 | 4.5 | 2.5 | 0.8 | 0.2 |

**Table S14.** Contribution (%) of environmental variables to the distribution of *Triplostegia* sp. A.

| Time | Bio18 | Bio11 | Bio3 | Bio15 | Bio2 | Bio7 |
| --- | --- | --- | --- | --- | --- | --- |
| Last Interglacial | 38.1 | 24 | 22.4 | 10.5 | 4.2 | 0.7 |
| Last Glacial Maximum | 37 | 25.4 | 23.3 | 10.7 | 3.4 | 0.2 |
| Mid Holocene | 38.4 | 24.3 | 23 | 11.2 | 2.6 | 0.4 |
| Current | 37.6 | 25.8 | 23.4 | 10.9 | 2.2 | 0.2 |
| 2090s SSPs 2-4.5  2090s SSPs 5-8.5 | 38.3 | 23.7 | 23.9 | 10.3 | 3.3 | 0.5 |
|  | 39.9 | 24.4 | 22.1 | 10.7 | 2.7 | 0.2 |

**Table S15.** Changes of suitable habitat area for *T. glandulifera*, *Triplostegia* sp. A, and *T. grandiflora* in different time periods and future climate change scenarios.

| Time periods | Area gained ( × 10^4^km^2^) | | | |  | Area lost ( × 10^4^km^2^) | | |  | Area change ( × 10^4^km^2^) | | |
| --- | --- | --- | --- | --- | --- | --- | --- | --- | --- | --- | --- | --- |
|  | GL | SP | GR |  | | GL | SP | GR |  | GL | SP | GR |
| LIG-Current | 99.38 | 46.59 | 46.4 |  | | 0 | 0 | 0 |  | 99.38 | 46.59 | 46.4 |
| LGM-Current | 101.09 | 46.61 | 46.4 |  | | 0 | 0 | 0 |  | 101.09 | 46.61 | 46.4 |
| MH-Current | 100.76 | 46.61 | 46.4 |  | | 0 | 0 | 0 |  | 100.76 | 46.61 | 46.4 |
| Current-Future (SSP2-4.5) | 25.74 | 4.35 | 5.37 |  | | 45.14 | 10.84 | 14.5 |  | -19.4 | -6.49 | -9.13 |
| Current-Future (SSP5-8.5) | 45.34 | 9.2 | 6.86 |  | | 62.88 | 18.57 | 27.65 |  | -17.54 | -9.37 | -20.79 |

Note: LIG: Last Interglacial; LGM, Last Glacial Maximum; MH, Mid Holocene; GL, *T. glandulifera*; SP, *Triplostegia* sp. A; GR, *T. grandiflora.*

**Table S16.** Niche overlap between *Triplostegia* species measured using Warren’s *I* and Schoener’s *D* indices in different time periods.

| Niche overlap index | Pairwise species |  |  |  | Time period | | |  |
| --- | --- | --- | --- | --- | --- | --- | --- | --- |
|  |  | LI | LGM | MH | Current | 2090s SSP2-4.5 | 2090s  SSP5-8.5 | |
| Schoener’s *D* | *T. glandulifera & T. grandiflora* | 0.07 | 0.10 | 0.19 | 0.46 | 0.47 | 0.37 | |
|  | *T. glandulifera & Triplostegia sp.* A | 0.11 | 0.14 | 0.12 | 0.51 | 0.52 | 0.48 | |
|  | *T. grandiflora & Triplostegia sp.* A | 0.14 | 0.27 | 0.33 | 0.60 | 0.62 | 0.59 | |
| Warren’s *I* | *T. glandulifera & T. grandiflora* | 0.17 | 0.23 | 0.36 | 0.74 | 0.73 | 0.64 | |
|  | *T. glandulifera & Triplostegia sp.* A | 0.37 | 0.41 | 0.38 | 0.80 | 0.79 | 0.76 | |
|  | *T. grandiflora & Triplostegia sp.* A | 0.35 | 0.53 | 0.62 | 0.86 | 0.87 | 0.84 | |

Note: LI, Last Interglacial; LGM, Last Glacial Maximum; MH, Middle Holocene; current, 1970-2000.





**Figure S1.** Map of the chloroplast genomes of the *Triplostegia* species. Genes located outside the circle are transcribed in a counter-clockwise direction, while those inside are transcribed clockwise. Genes belonging to different functional groups are color-coded following the legend. The dark shading in the inner circle indicates GC content. LSC represents large single copy, SSC represents small single copy, and IR represents inverted repeat regions.

**
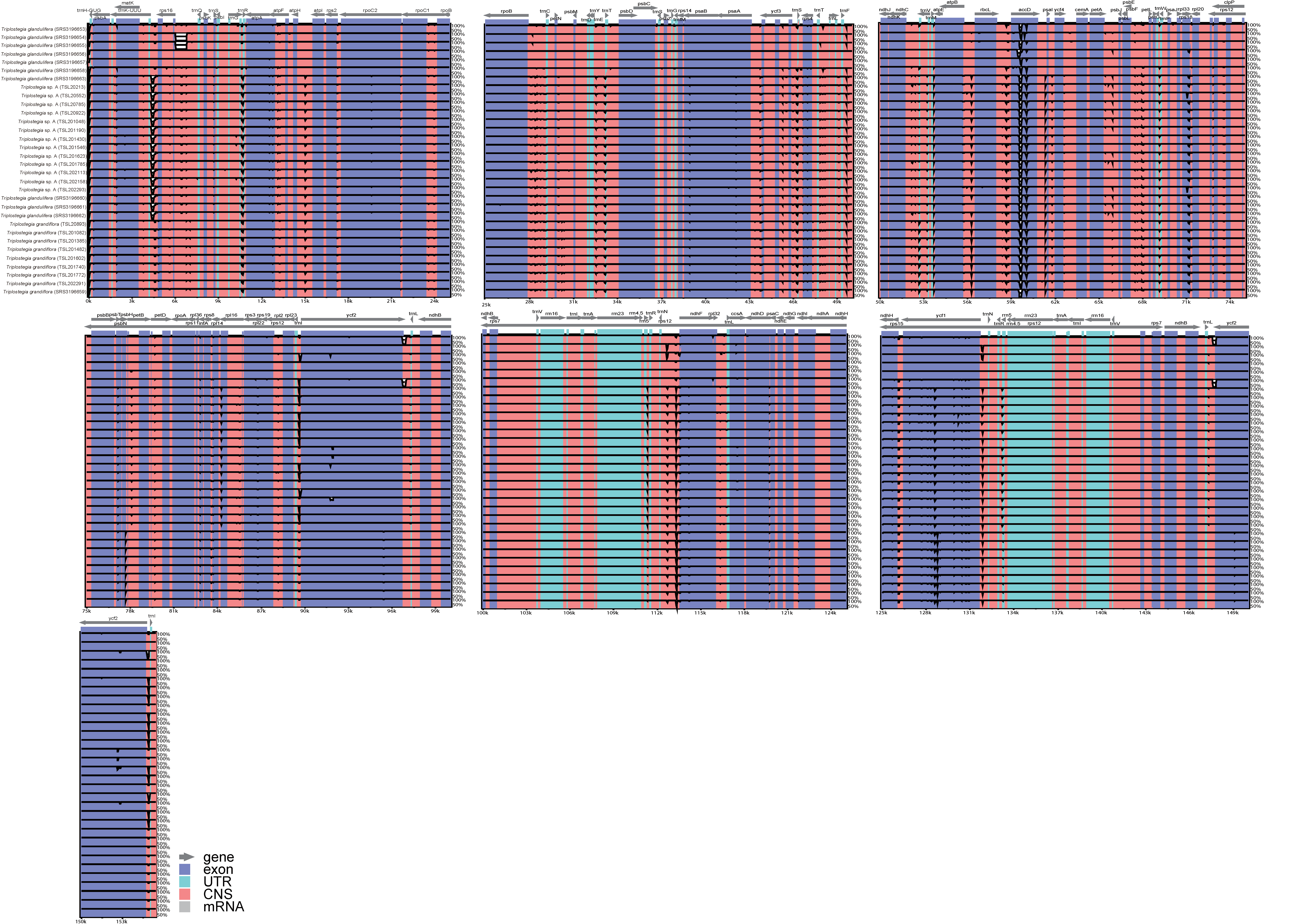
**

**Figure S2.** Comparison of plastid genomes among 33 *Triplostegia* samples using mVISTA under Shuffle-LAGAN mode. Gray arrows above the alignment indicate the gene direction. The dark blue regions represent exons, the light-blue regions represent untranslated regions (UTRs), and the pink regions represent conserved non-coding sequences (CNS). The y-axis represents the percent identity ranging from 50% to 100%.





**Figure S3.** Comparison of complete chloroplast genomes among 33 *Triplostegia* samples using MAUVE algorithm.





**Figure S4.** Comparative analysis of chloroplast genome boundaries among 33 samples of *Triplostegia.*

**
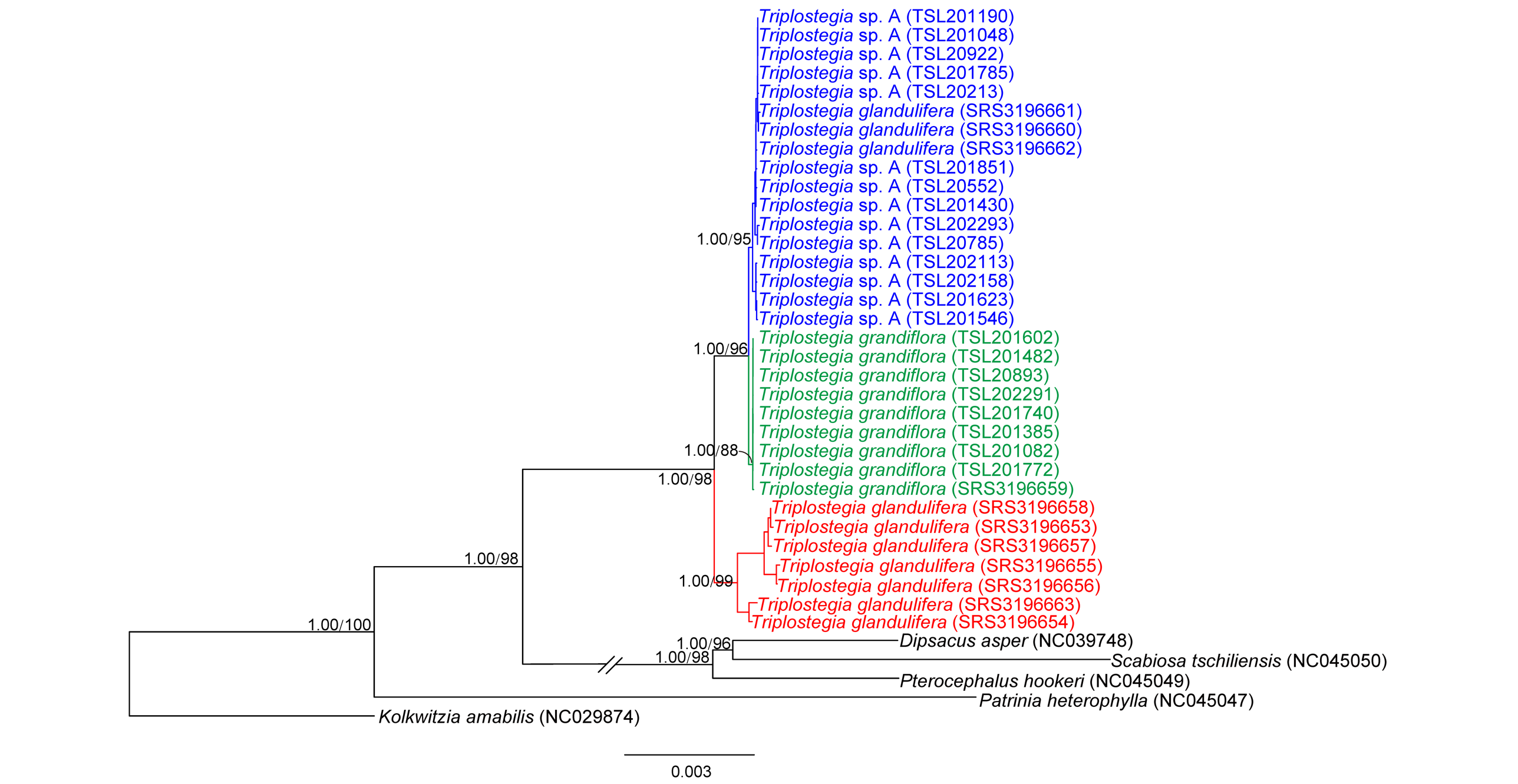
**

**Figure S5.** Phylogenetic relationships of 33 samples of *Triplostegia* species based on the plastid CDS dataset. The phylogenetic tree was constructed using both maximum likelihood (ML) and Bayesian inference (BI) methods. The maximum likelihood (ML) tree is presented. Numbers along the branch indicate bootstrap support values from ML analysis (based on 1000 replicates) and Bayesian posterior probabilities from BI analysis.


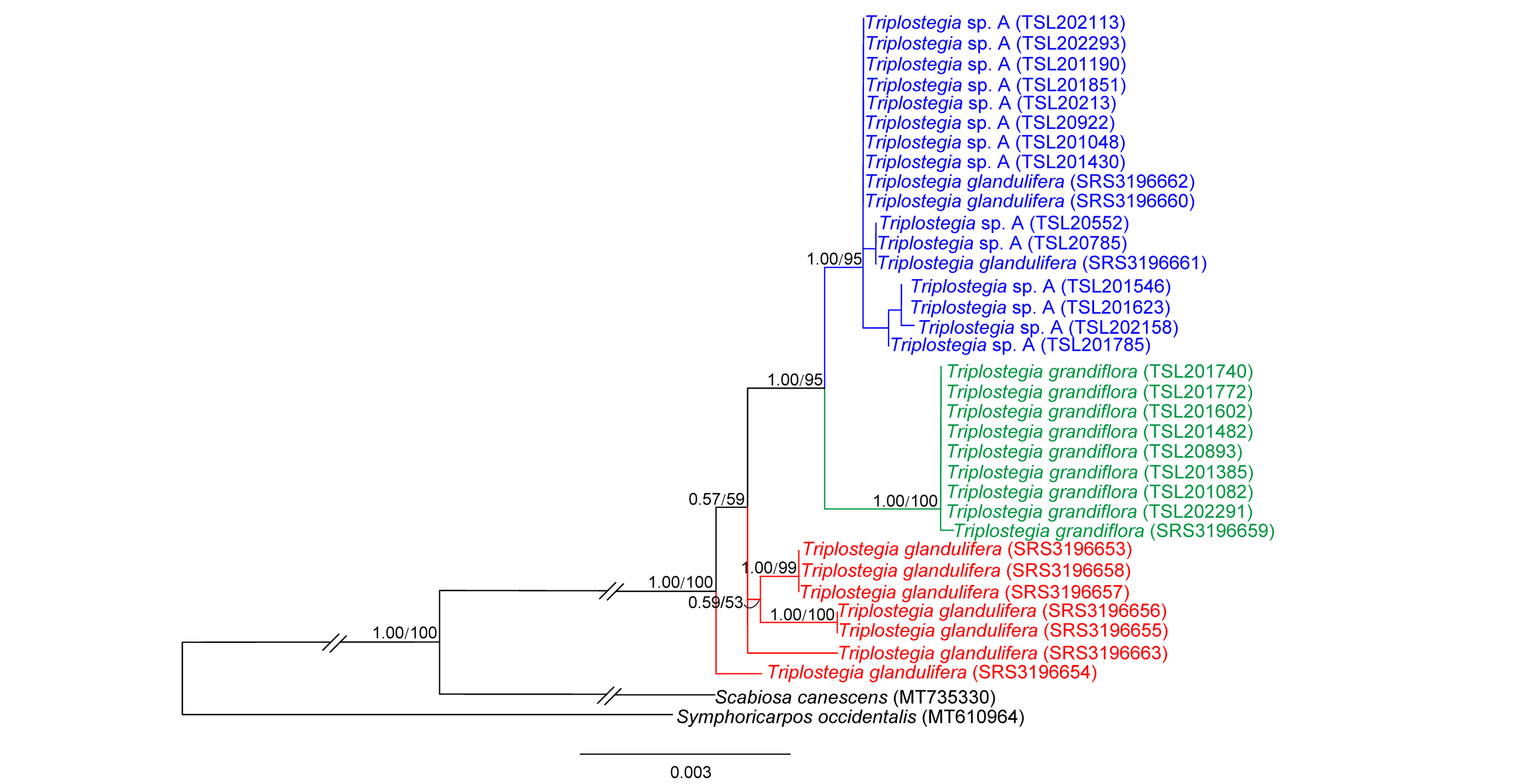


**Figure S6.** Phylogenetic relationships of 33 samples of *Triplostegia* species based on the nrDNA sequences. The phylogenetic tree was constructed using both maximum likelihood (ML) and Bayesian inference (BI) methods. The maximum likelihood (ML) tree is presented. Numbers along the branch indicate bootstrap support values from ML analysis (based on 1000 replicates) and Bayesian posterior probabilities from BI analysis.

**
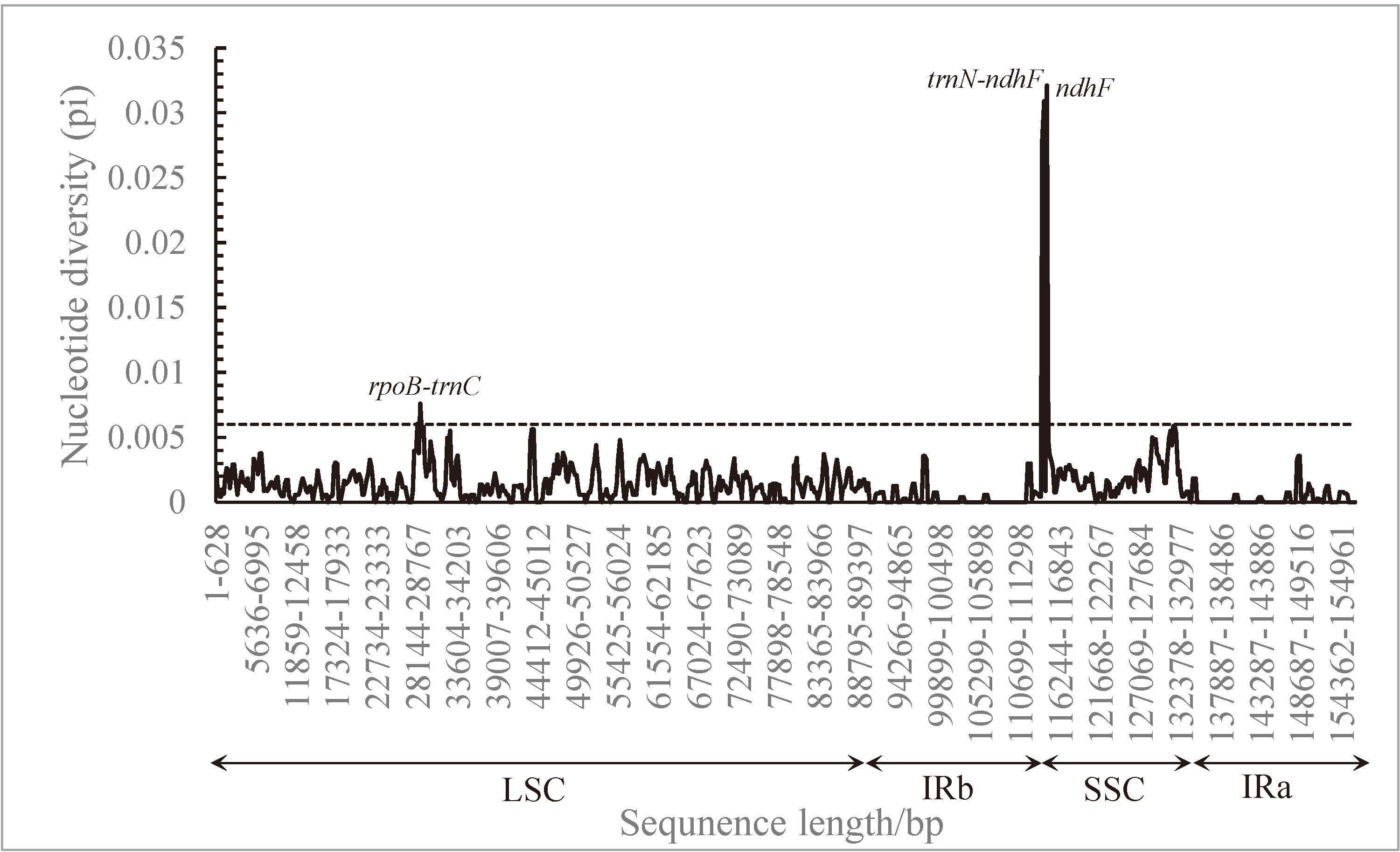
**

**Figure S7.** Sliding window analysis of the 33 chloroplast genomes of *Triplostegia*. The X-axis represents the position of the midpoint of a window, while the Y-axis represents the nucleotide diversity (Pi) of each window. Mutational hotspots and highly divergent loci are marked.


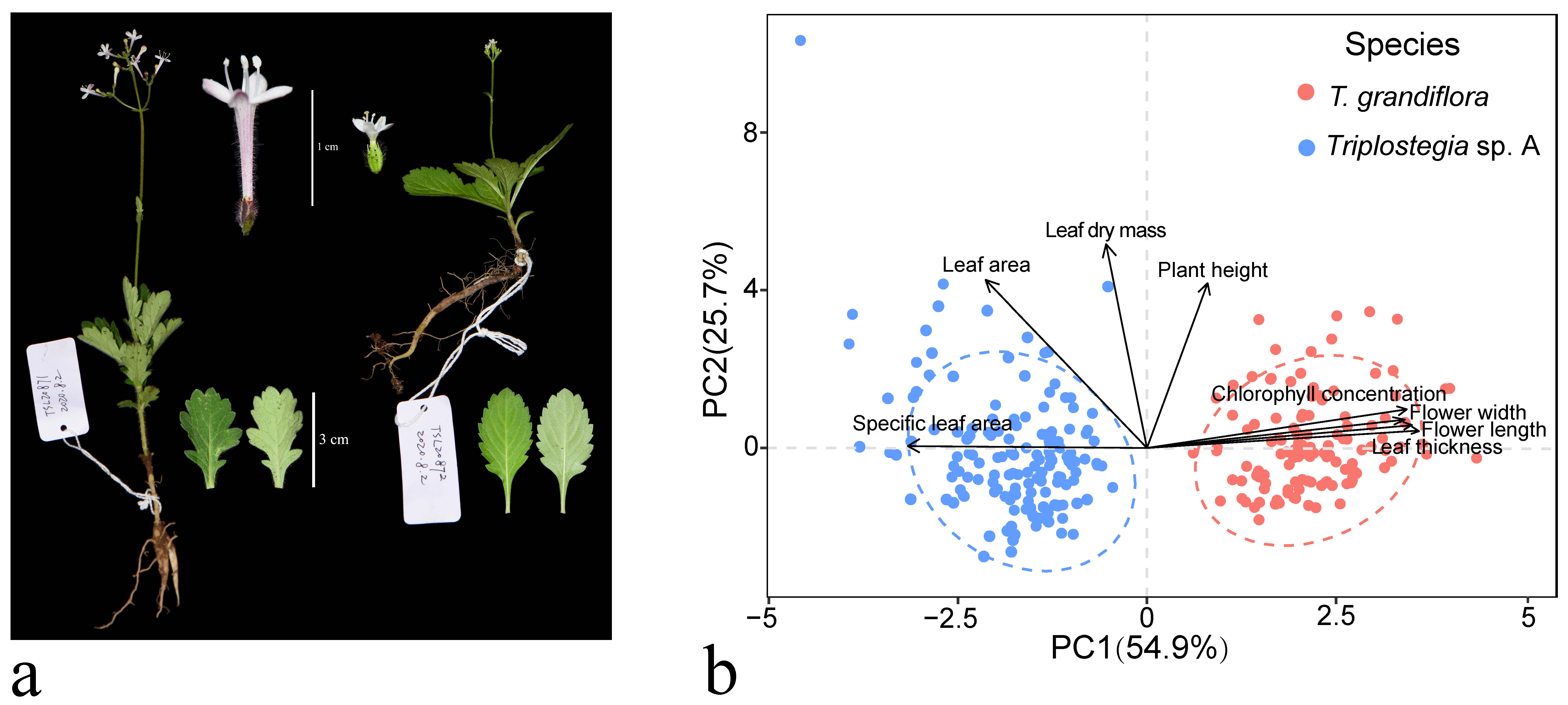


**Figure S8.** (a) Morphology comparison of *T. grandiflora* (left) and *Triplostegia* sp. A (right) co-distributed in northwestern Yunnan, the Hengduan Mountains Region. (b) Principal component analysis (PCA) of eight functional traits of *T. grandiflora* and *Triplostegia* sp. A co-distributed in northwestern Yunnan.

**
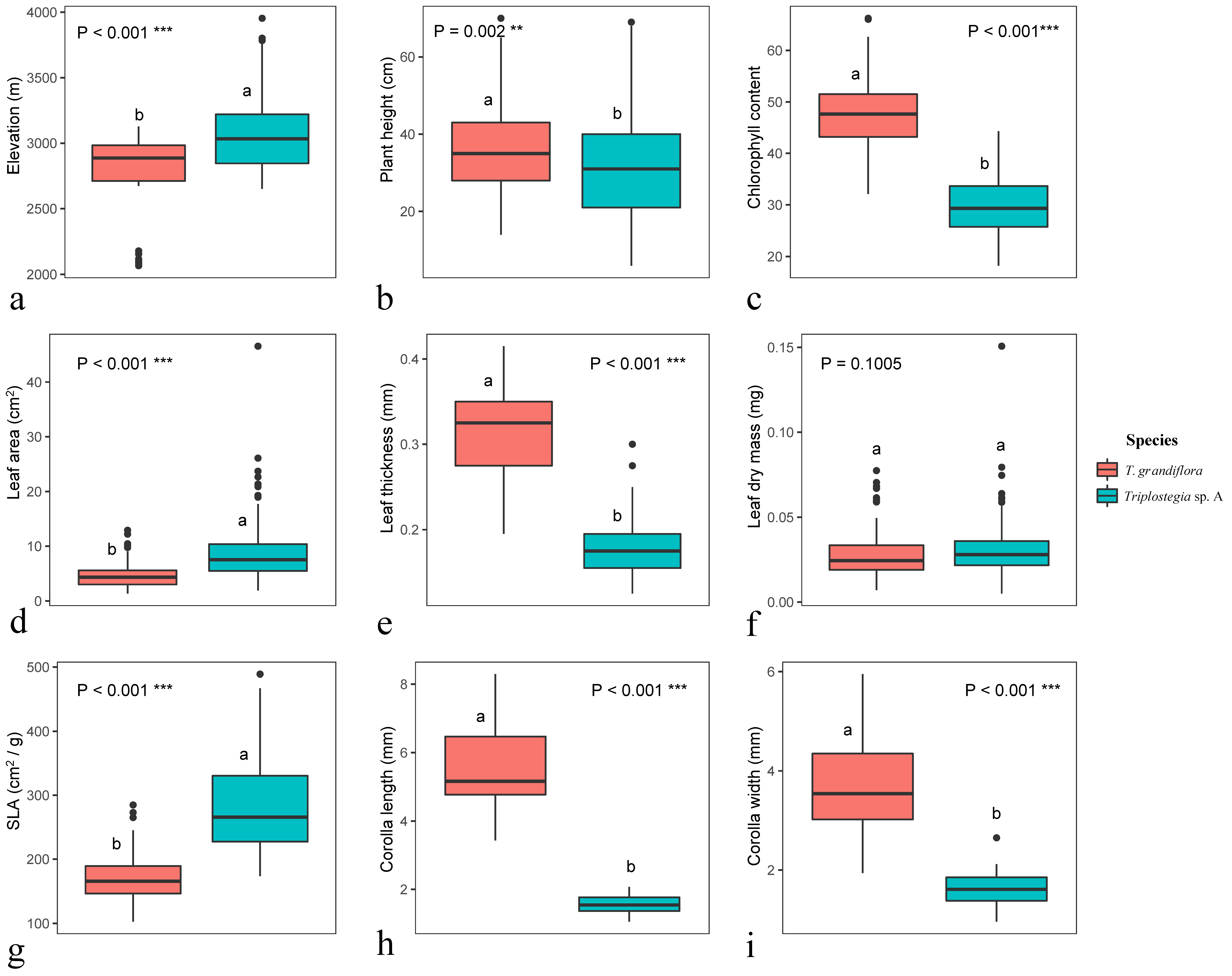
**

**Figure S9.** Box plot depicting the elevation ranges (a) and functional traits (b-i) of *T. grandiflora* and *Triplostegia* sp. A co-distributed in northwestern Yunnan.


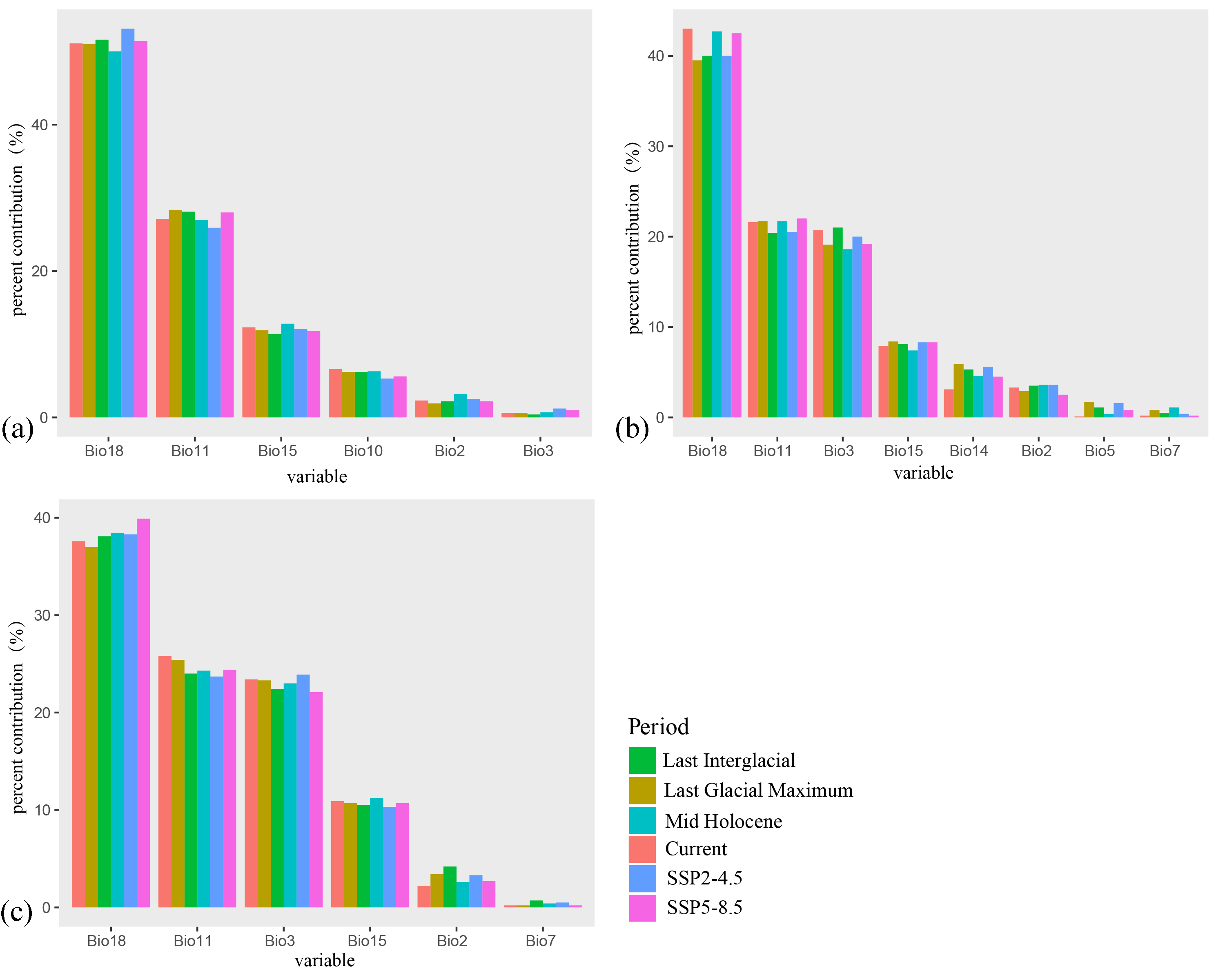


**Figure S10.** Bar plot illustrating the percent contribution of climatic variables to the distribution of *T. glandulifera* (a), *T. grandiflora* (b), and *Triplostegia* sp. A (c) in different time periods and future climate scenarios.


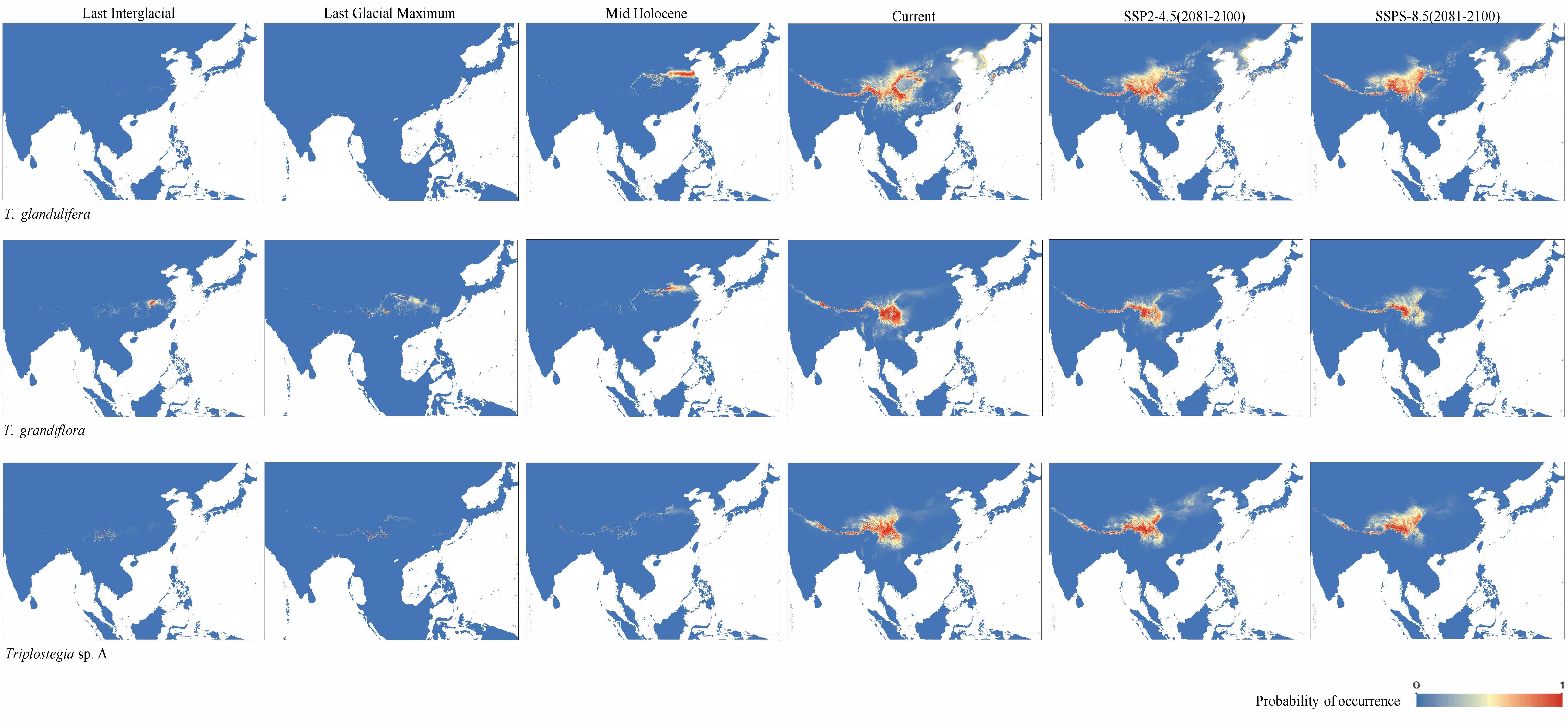


**Figure S11.** Distribution of suitable areas for *T. glandulifera*, *T. grandiflora* and *Triplostegia* sp. A in different time periods and future climate change scenarios. (The maps are created by authors using ArcGIS software)





**Figure S12.** Changes in suitable habitat for *T. glandulifera* (a, SSP2-4.5; b, SSP5-8.5; c, bar plot of range changes), *T. grandiflora* (d, SSP2-4.5; e, SSP5-8.5; f, bar plot of range changes), and *Triplostegia* sp. A (g, SSP2-4.5; h, SSP5-8.5; i, bar plot of range changes) under two future climate scenarios. The red area represents potential range contraction, blue area represents potential range expansion, and green area represents overlap between current and projected future ranges. In the bar plots, red indicates area contraction, and blue indicates area expansion. (The maps are created by authors using ArcGIS software)
